# Mechanistic insights into enterocin C targeting the undecaprenyl phosphate recycling protein BacA

**DOI:** 10.1016/j.jbc.2025.110929

**Published:** 2025-11-11

**Authors:** Victor Folcher, Rodolphe Auger, Clara Louche, Pascale Serror, Thierry Touzé

**Affiliations:** 1 Université Paris-Saclay, CEA, CNRS, Institute for Integrative Biology of the Cell (I2BC), Gif-sur-Yvette, France; 2 Université Paris-Saclay, INRAE, AgroParisTech, Micalis Institute, Jouy-en-Josas, France; 3 Institut Hospitalo-Universitaire Comprehensive SEPSIS Center, Paris-Saclay University, Saclay, France

**Keywords:** bacteriocins, *Enterococcus faecalis*, membrane, undecaprenyl phosphate, membrane protein

## Abstract

*Enterococcus faecalis* is an important opportunistic pathogen responsible for healthcare-associated infections. It is intrinsically resistant to various antibiotics, particularly to cephalosporins and vancomycin, creating an urgent need for alternative therapeutics. In this context, bacteriocins warrant investigations as a potential source of medically useful antibiotics. Herein, we demonstrate that Enterocin C, a class IIb two-peptide bacteriocin, specifically targets the membrane-embedded undecaprenyl phosphate recycling protein BacA from enterococci as a cell surface receptor. Using biochemical and biophysical methods, supported by computer modeling and mutagenesis, we deciphered the EntC's molecular interaction pattern with its target, marking the first mechanistic insight of a two-peptide bacteriocin. The two peptides act cooperatively at nanomolar concentrations to interact with the outward-open catalytic pocket of BacA: the peptide EntC1 docks deeply into the catalytic site, inhibits BacA's enzymatic activity, and enables the binding of peptide EntC2, eliciting membrane permeabilization, eventually leading to cell death. This work paves the way for the bioengineering of BacA-targeting bacteriocins to develop tailored antimicrobial strategies.

The emergence of multidrug-resistant (MDR) and extensively drug-resistant (XDR) bacterial strains is one of the top 10 most critical global health concerns, according to the World Health Organization (WHO), stressing the urgent need for therapeutic alternatives (1). The pathogenic bacteria responsible for life-threatening nosocomial infections and developing antibiotic resistance are collectively termed “ESKAPE pathogens”, which encompass *Enterococcus* sp. (*Enterococcus faecium* and *Enterococcus faecalis*), *Staphylococcus aureus*, *Klebsiella pneumoniae*, *Acinetobacter baumannii*, *Pseudomonas aeruginosa*, and *Enterobacter* species (2). In response to this escalating antibiotic resistance crisis, bacteriocins are emerging as promising alternatives. These antimicrobial peptides are produced by bacteria across all phyla through ribosomal synthesis (3). They are widespread in nature and exhibit diversity in their structures and mechanisms of action. They show a narrow to broad inhibitory activity and are featured by high antibacterial activities, causing bacterial cell death at nanomolar to picomolar concentrations, with minimal or no cytotoxicity effects on eukaryotic cells. Despite their numerous advantages, only a few bacteriocins have progressed beyond preclinical trials, and none are currently employed in human therapy, which can be explained by a lack of understanding of their mechanisms of action. Elucidating these mechanisms and their specificity is therefore critical in selecting them for the treatment of antibiotic-resistant bacterial infections.

Bacteriocins are categorized into two classes based on whether their founding peptides undergo post-translational modifications (class I) or not (class II) (3). Class IIb bacteriocins comprise two linear peptides that permeabilize cell membranes of target cells through unknown mechanisms (4, 5). Class IIb bacteriocins include four homologous bacteriocins, Lactococcin G (LcnG), lactococcin Q (LcnQ), Enterocin 1071 (Ent1071), and Enterocin C (EntC) (Fig. 1*A*), among at least 15 bacteriocins. Class IIb bacteriocins act *via* a cell surface receptor-mediated process conferring them high specificity of action. LcnG, produced by *Lactococcus lactis,* consists of peptides LcnG_α_ and LcnG_β_. While these peptides are ineffective individually, they exhibit antibacterial activity at nanomolar concentrations against *L. lactis* strains when combined in a 1:1 ratio, causing membrane permeabilization to monovalent cations (6, 7, 8). Whole-genome sequencing of LcnG-resistant clones revealed stop codons or frameshift mutations in the *bacA* gene indicating that BacA may constitute its cell surface receptor (9). BacA is a membrane-embedded protein, which is widely conserved in bacteria. It is involved in the recycling of the undecaprenyl phosphate (C_55_-P), a lipid carrier enabling peptidoglycan subunit translocation across the plasma membrane (Fig. S1) (10). When bound to the peptidoglycan disaccharide-pentapeptide subunit, C_55_-P forms the lipid II membrane intermediate. Following the subunit transfer to the nascent peptidoglycan, the lipid carrier is released as undecaprenyl pyrophosphate (C_55_-PP), which is recycled to sustain peptidoglycan, and other cell wall glycopolymers, biosynthesis (11). The recycling consists of C_55_-PP dephosphorylation followed by the flip of C_55_-P to the inner side of the membrane. Either BacA or a protein from the PAP2 superfamily catalyze C_55_-PP dephosphorylation (12) (Fig. S1). According to its crystal structure, which resembles that of transporter proteins, BacA was hypothesized to also catalyze the flip of C_55_-P (13, 14). Recent studies have identified additional membrane proteins with C_55_-P flippase activity, including members of the DedA family and DUF368-containing proteins (15, 16). In the present study, we have investigated the mechanism of action of Enterocin C (EntC), an LcnG homolog produced by *E. faecalis* C901 strain (Fig. 1*A*). EntC, composed of EntC1 and EntC2 peptides, is active against the opportunistic pathogen *E. faecalis* (17). We demonstrated that EntC relies on BacA to exert its antibacterial activity and its binding mode to BacA was investigated in depth using complementary *in vitro* approaches from which, in combination with computer modelling and mutagenesis, we could infer a model of action. Our findings demonstrate that EntC1 binds to the outward-open catalytic pocket of BacA, inhibiting its enzymatic activity and enabling the further binding of EntC2 in a cooperative manner. This latter interaction stabilizes the tripartite complex and triggers membrane permeabilization likely by anchoring the peptides, as one functional unit, deeply into the lipid bilayer, ultimately leading to cell death. We herein describe, for the first time, the binding mode of a two-peptide bacteriocin to its membrane-embedded target paving the way for the further development and bioengineering of this class of bacteriocins for narrow-spectrum antibiotics with minimal impact on microbiota.

**Figure 1:**
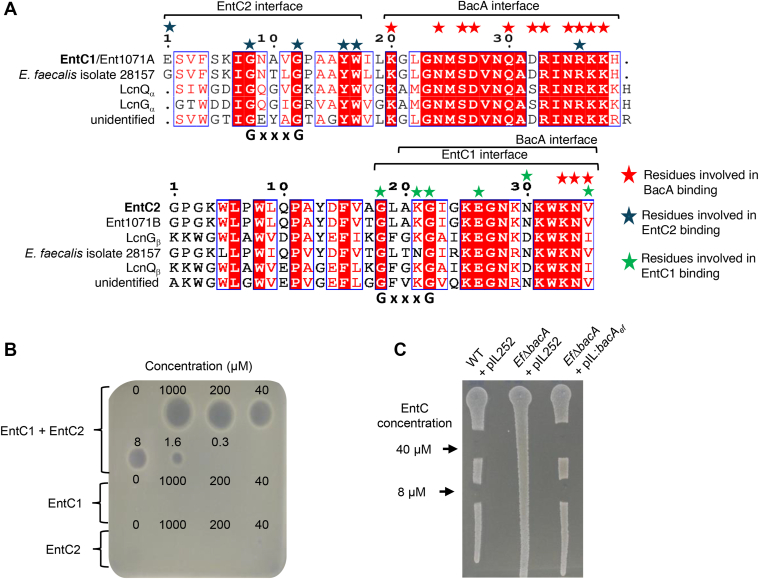
**Sequence alignment and activity measurement of EntC.***A*, sequence alignment of EntC-related peptides. The interface regions and residues involved in binding, according to AlphaFold2 models, are indicated above the sequences (*red stars* indicate residues involved in BacA binding, *blue stars* for EntC2 binding and *green stars* for EntC1 binding). *Blue boxes* highlight similar sequences, and identical sequences are filled in *red*. The GxxxG motif common to all aligned peptides is highlighted below the sequences. *B*, dose effect of EntC peptides on *E. faecalis* WT strain. 6 μl-droplets of peptide solutions at the indicated concentrations are spotted on a lawn of *E. faecalis* cells. Results are representative of three independent experiments. *C*, activity measurement of EntC on WT and *Ef*Δ*bacA* cells carrying plasmids pIL252 (empty vector) or pIL:*bacA*_*ef*_. The indicated cells were patched on a BHI agar plate, and a drop of EntC solution at the indicated concentration was spotted. Results are representative of three independent experiments.

## Results

### BacA is targeted by EntC peptides

To study the mechanism of action of EntC, EntC1 and EntC2 mature peptides, *i.e.* without their N-terminal signal sequence (Fig. 1*A*), were obtained by chemical synthesis. Spot-on lawn assays demonstrate the susceptibility of the clinical *E. faecalis* vancomycin-resistant V583 strain to the synthetic peptides when they were combined (Fig. 1*B*). In contrast, neither peptide exhibited antibacterial activity when applied individually. The concentration of EntC required to prevent the growth of *E. faecalis* cells was determined in different liquid culture media showing varying values: 100 nM in BHI, 50 nM in M17, and 2 nM in MRS (Fig. S2). This finding aligns with previous reports according to which growth in MRS medium optimizes bacteriocins efficacy in comparison to other media for a yet unknown reason (18, 19). To establish whether BacA is the target of EntC, the *bacA*-deleted strain, *Ef*Δ*bacA*, was generated. The latter strain displayed a similar growth phenotype as the wild-type strain but exhibited a full resistance to EntC (Fig. 1*C*). The susceptibility of *Ef*Δ*bacA* strain to EntC was fully restored upon the expression of an ectopic copy of *bacA* (Fig. 1*C*). Our data demonstrate that the peptides exert a synergistic antibacterial activity against *E. faecalis* that is fully dependent on BacA protein.

### EntC inhibits the enzymatic activity of BacA_*ef*_

**Figure 2:**
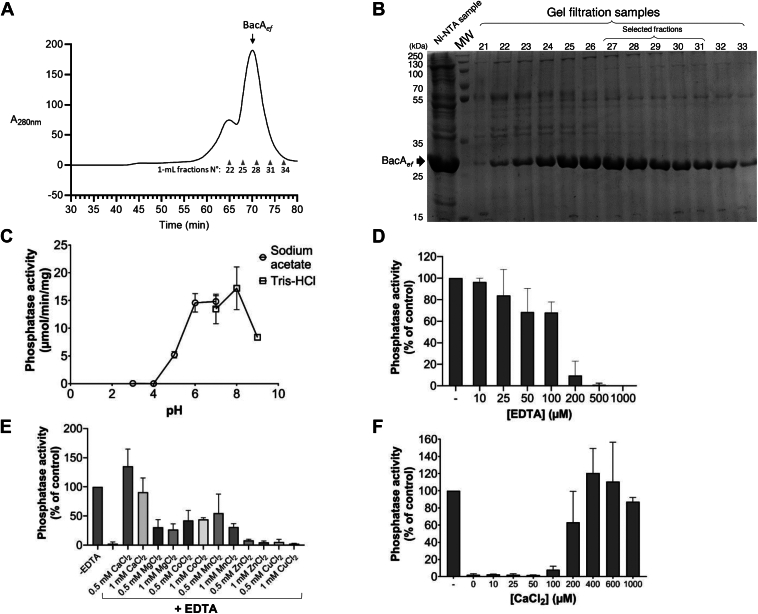
**Purification and biochemical characterization of BacA_*ef*_.***A*, Gel filtration chromatogram on a Superdex 200 resin of BacA_*ef*_ sample protein obtained after affinity purification on Ni^2+^-NTA-agarose. Elution was performed at a flow rate of 1 ml/min, coupled with UV spectrophotometry at 280 nm measured at the column outlet. The collected samples are indicated with their corresponding numbers. *B*, SDS-PAGE analysis of 10 μl protein samples obtained from affinity purification and gel filtration chromatography. The samples were prepared in 1x Laemmli buffer and separated on a 12% acrylamide gel. Proteins were revealed using Coomassie Blue staining. The molecular weight (MW) marker is indicated on the *left* and BacA_*ef*_ is indicated by a *black arrow*. The samples that were selected for further studies were pooled and concentrated up to 1 mg/ml, using a VivaSpin filter, before their storage at −20 °C. *C*, BacA_*ef*_ C_55_-PP phosphatase activity measured at various pH. Activity measurements were performed in sodium acetate buffer for pH ranging from 3 to 7 (*round dots*), and Tris-HCl buffer for pH ranging from 7 to 9 (*square dots*). *D*, dose-dependent effect of EDTA on BacA_*ef*_ C_55_-PP phosphatase activity. The activity was measured at pH 7.5 in Tris-HCl buffer in the presence of 0.1% DDM. *E*, restoration of BacA_*ef*_ activity by different divalent cations following treatment with 500 μM EDTA. *F*, dose-dependent effect of CaCl_2_ on the activity of EDTA-treated BacA_*ef*_. Activity was measured at pH 7.5 in Tris-HCl buffer with 0.1% DDM.

**Figure 3:**
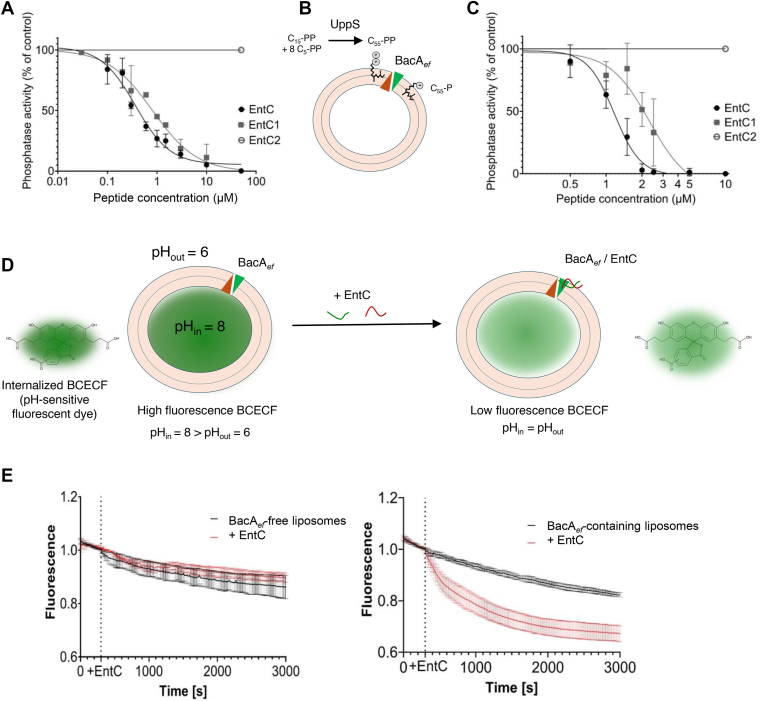
**Effect of EntC on BacA_*ef*_ C_55_-PP phosphatase activity and membrane integrity.***A*, dose effect of EntC peptides on BacA C_55_-PP phosphatase activity in DDM micelles. *B*, schematic representation of UppS-coupled C_55_-PP phosphatase assay in liposome. *C*, dose effect of EntC peptides on BacA C_55_-PP phosphatase activity in liposome. *D*, schematic representation of the permeabilization assay using the BCECF pH sensitive dye. BCECF is internalized in the lumen of liposomes at internal pH = 8 and liposomes are dispersed in buffer at pH = 6 to assess membrane integrity over time before and after the addition of EntC. *E*, membrane permeabilization activity of EntC. Fluorescence measurements are normalized to values recorded prior to the addition of 5 μM EntC (*dashed line*). The panel shows results obtained with BCECF-containing liposomes without (*left panel*) or with BacA_*ef*_ reconstituted into the lipid bilayer (*right panel*). The membrane integrity is followed over time before and after the addition of EntC (*red lines*) or without treatment (*black lines*). Results are the average of three independent experiments and error bars indicate standard deviations.

To investigate the mode of binding of EntC to BacA, an N-terminal His_6_-tagged BacA from *E. faecalis* (BacA_*ef*_) was produced in *Escherichia coli,* solubilized in *n*-dodecyl-β-D-maltoside (DDM) detergent and purified by affinity purification followed by gel filtration (Fig. 2, *A* and *B*). To assess the functionality of the purified BacA_*ef*_, its C_55_-PP phosphatase activity was measured. The optimal pH for BacA_*ef*_ was determined between pH 6 and 8 (Fig. 2*C*), and the presence of EDTA inhibited its activity (Fig. 2*D*). The activity was restored upon the addition of CaCl_2_ following EDTA treatment, and to a lesser extent by MgCl_2_, MnCl_2_ and CoCl_2_ (Fig. 2, *E* and *F*). The C_55_-PP phosphatase activity of BacA_*ef*_ was determined to be 27.0 ± 4.6 μmol/min/mg in the optimal conditions (*i.e.* 20 mM Tris-HCl, pH 7.4, 400 μM CaCl_2_, 150 mM NaCl, 0.1% (w/v) DDM, and 50 μM C_55_-PP) (Table S1). The BacA from *E. coli* (BacA_*ec*_) was also purified, and its C_55_-PP phosphatase activity was measured in the same conditions as for BacA_*ef*_, providing an activity of 9.1 ± 0.6 μmol/min/mg (Table S1). EntC (*i.e.* EntC1 and EntC2 in 1:1 M ratio) and EntC1 alone exhibited a dose-dependent inhibition of BacA_*ef*_ enzymatic activity (Fig. 3*A* and Table S1). The concentration required to reduce the activity by 50%, or half maximal inhibitory concentration, IC_50_ value, was found to be 0.4 ± 0.1 μM with EntC and 0.8 ± 0.2 μM with EntC1. In contrast, no inhibition of BacA_*ef*_ was observed in the presence of EntC2 alone up to 50 μM in the reaction mixture (Fig. 3*A*). These data demonstrate that EntC1 directly binds BacA_*ef*_ and blocks its catalytic cycle. The decrease in the IC_50_ value observed in the presence of both peptides (*i.e.* EntC) compared to EntC1 alone (*p-*value = 0.0007), suggests that EntC2 cooperates with EntC1 to enhance or facilitate BacA binding. The activity of BacA_*ec*_, which shares 41% sequence identity with BacA_*ef*_ (Fig. S3), was not inhibited by EntC (Table S1), highlighting a high specificity.

To assess EntC activity towards BacA_*ef*_ in a membrane-like environment, the latter was reconstituted in liposomes. A coupled enzymatic assay was developed using *E. coli* UppS (C_55_-PP synthase) and water-soluble C_55_-PP precursors in order to synthesize C_55_-PP in the liposome-containing reaction mixture and to allow its partition in the lipid bilayer (Fig. 3*B*). Using BacA-free liposomes, the synthesis of C_55_-PP was observed upon the addition of UppS and C_55_-PP precursors. A fraction of this C_55_-PP was further converted into C_55_-P with BacA-containing liposomes only, assessing the proper folding of BacA in the lipid bilayer (Table S1). A dose-dependent inhibition of BacA_*ef*_ by EntC and EntC1 was observed with IC_50_ values of 1.2 ± 0.1 μM and 2.2 ± 0.3 μM, respectively, while EntC2 had no effect (Fig. 3*C* and Table S1). At the highest concentration tested (100 μM), EntC had no inhibitory effect on the activity of BacA_*ec*_ when similarly reconstituted in liposomes (Table S1).

### EntC permeabilizes BacA_*ef*_-containing liposomes

To investigate whether EntC can disrupt lipid bilayers in a BacA-dependent manner, we used a pH-sensitive non-permeant fluorescent dye 2′,7′-bis(2-carboxyethyl)-5-carboxy-fluorescein, BCECF, that was internalized in the lumen of liposomes at internal pH 8 (see Materials and Methods). Then, BCECF fluorescence was monitored over time after diluting the liposomes into a lower-pH buffer (pH 6) (Fig. 3*D*). BacA-liposomes and protein-free liposomes displayed the same slow decrease of BCECF fluorescence over time in the absence of EntC (1.10^-4^ sec^-1^) (Fig. 3*E*). The addition of 5 μM of EntC to BacA_*ef*_-liposomes (peptide to lipid molar ratio, c.a. 1:1300) strongly enhanced the quenching of BCECF by the lower external pH (decrease rate about 6.10^-4^ sec^-1^), while it did not affect protein-free liposomes (Fig. 3*E*). In order to determine whether the presence of any membrane protein would sensitize the liposomes to EntC, we also tested PgpB-liposomes. PgpB is a PAP2 C_55_-PP phosphatase from *E. coli* with a completely different fold and catalytic mechanism as compared to BacA (13, 14, 20). In this case, EntC had no permeabilization effect (Fig. S4). These results indicate that EntC dissipates the pH gradient across the membrane of BacA_*ef*_-containing liposomes only, supporting that BacA is essential and sufficient for EntC to elicit the permeabilization of target cell membranes.

### EntC peptides form a complex with BacA_*ef*_ in 1:1 stoichiometry

**Figure 4:**
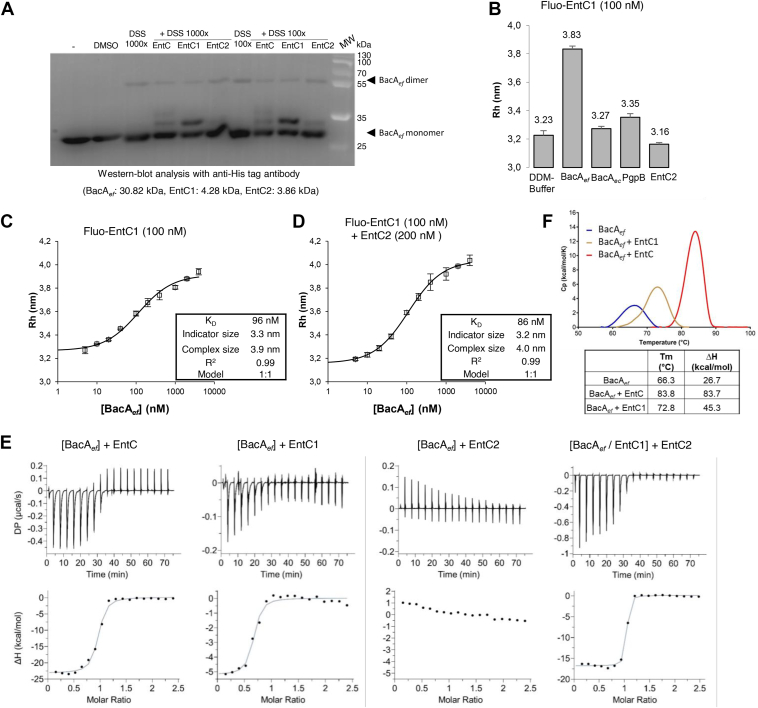
**Interaction measurement between BacA_*ef*_ and EntC peptides.***A*, DSS Crosslinking of BacA_*ef*_ sample in the absence and in the presence of EntC peptides added at equimolar concentrations (17 μM) at two different protein to DSS ratios (100- or 1000-fold excess). *B*, determination of hydrodynamic radii (Rh) of Fluorescein-labelled EntC1 by Flow-Induced Dispersion Analysis (FIDA). Fluo-EntC1 was dispersed in DDM-containing buffer with or without proteins or EntC2 peptide. Results are the average of three independent experiments, and error bars indicate standard deviations. *C* and *D*, FIDA determination of the dissociation constant of Fluo-EntC1 towards BacA_*ef*_ in the absence (*C*) and in the presence of EntC2 (*D*). Results are the average of three independent experiments, and error bars indicate standard deviations. *E*, Isothermal Titration Calorimetry (ITC) scans for BacA_*ef*_ titration with EntC peptides. *Upper panels*, ITC thermogram responses for the titration of EntC peptides (400 μM) into the indicated mixture, indicated between brackets (33 μM). *Lower panels*, heat profile from peak integration of the ITC thermograms. The data shown are representative of experiments performed at least in triplicate. *F*, stability enhancement of BacA_*ef*_ by EntC peptides. *Upper panel*, representative DSC thermograms of BacA_*ef*_ (33 μM) in the absence and in the presence of EntC peptides (66 μM). *Lower table*, Tm and thermodynamic parameters. DSC scans were performed at 60 °C/h between 30 °C and 100 °C. The data shown are representative of experiments performed in triplicate.

To highlight the formation of complexes between EntC peptides and BacA_*ef*_, *in vitro* crosslinking experiments were performed using the amine-reactive, membrane-permeable and non-cleavable crosslinker disuccinimidyl suberate (DSS). The peptides and BacA were incubated at equimolar concentrations (*i.e.* 17 μM) in the presence of a molar excess of DSS. The crosslinking reaction was quenched, the mixture was resolved by SDS-PAGE and BacA was revealed by western blotting. As shown in Figure 4*A*, in the presence of EntC and DSS, two faint bands appeared above the BacA monomer, which likely correspond to BacA complexed with one and two peptides, respectively, according to their apparent molecular mass. In the presence of EntC1 alone, a strong and unique band was observed above the BacA monomer with a molecular mass corresponding to a BacA_*ef*_:EntC1 complex with a 1:1 stoichiometry (Fig. 4*A*). When EntC2 was added alone, a very faint band was also observed above BacA, slightly below the BacA_*ef*_:EntC1 complex. According to the molecular mass of EntC2 (*i.e*., 3.86 kDa) compared to that of EntC1 (*i.e*., 4.82 kDa), the latter band likely corresponds to BacA_*ef*_:EntC2 complex. Notably, dimers of BacA were also observed at c.a. 60 kDa in the presence of DSS only (Fig. 4*A*). BacA_*ec*_ was previously found to crystallize as dimers by Workman *et al.*, but whether the dimers were biologically relevant was not determined (14). Herein, the fact that BacA_*ef*_ also dimerizes in DDM micelles supports a physiologically relevant oligomerization. Together, these results show that both peptides can bind BacA separately, but with a much greater propensity of binding for EntC1. The band corresponding to the covalent BacA_*ef*_:EntC1 complex was further analyzed by mass spectrometry after trypsin digestion. As expected, peptides from both BacA_*ef*_ and EntC1 were recovered, with 55% and 69% coverage, respectively (Fig. S5). Unfortunately, no cross-linked BacA_*ef*_:EntC1 peptide, that could have helped us to determine their interface, was recovered, presumably due to limited coverage.

### EntC1 binds BacA_*ef*_ with high affinity

Flow-Induced Dispersion Analysis (FIDA) is used to determine the hydrodynamic radius (Rh) of polypeptides based on the fact that the laminar flow profile of particles through narrow capillaries is governed by their Rh (21). The Rh is then determined from the measurement of the flow profile of a fluorescently labelled species. FIDA can be used to monitor protein-protein interaction through changes in the flow profile of the fluorescent species upon interaction with partners (≥10% increment of the Rh can be detected). To evaluate the BacA_*ef*_:EntC interaction, EntC peptides were then individually and randomly labeled at primary amino groups with fluorescein. Unfortunately, the labeling of EntC2 was not successful in contrast to EntC1 labeling (see Materials and Methods). The dispersion of fluorescein-EntC1 (Fluo-EntC1) at 100 nM in detergent-free solution yielded an Rh of 0.69 nm, and the peptide was found to adhere to the capillary as judged from the deviation of the Taylorgram from the ideal Gaussian shape expected for pure dispersion (Fig. S6). Upon dispersion of Fluo-EntC1 in a buffer containing 0.1% DDM, an Rh of 3.2 nm was yielded, and no adherence to the capillary was observed (Figs. 4*B* and S6). This size is likely representative of a particle corresponding to the peptide within a DDM micelle, as judged from the size increment in the presence of detergent, since there is an exponential relationship between diffusivity and molecular weight (22, 23). Previous studies using circular dichroism and NMR spectroscopy have revealed that both LcnG peptides were unstructured in water, but became structured upon exposure to micelles or liposomes (24, 25). Our results strongly suggest that EntC1 shares this feature. Upon dispersion of EntC1 in a buffer containing BacA_*ef*_ micelles (at 2 μM of BacA_*ef*_), an Rh of 3.8 nm was yielded, demonstrating the binding of EntC1 to BacA_*ef*_ micelles according to the significant size increment, while no significant change of EntC1 Rh was observed in the presence BacA_*ec*_ or PgpB micelles (Fig. 4*B*). The addition of EntC2 did not induce an increase of EntC1 Rh. However, due to the small size of the peptide compared to the size of the micelle, we could not infer whether EntC2 interacted with EntC1-containing micelles or not (Fig. 4*B*). We then measured the dispersion of Fluo-EntC1 upon titration with BacA_*ef*_ micelles (Fig. 4*C*). The titration curve was best fitted with a 1:1 binding model yielding a *K*_d_ of 96 nM. The addition of EntC2 in the latter experiment at twice the concentration of EntC1, displayed a similar binding curve, best fitted with 1:1 binding model, and yielded a *K*_d_ of 86 nM (Fig. 4*D*).

### EntC peptides bind cooperatively with BacA_*ef*_

As EntC2 binding parameters could not be determined by FIDA, the binding was also assessed in solution by Isothermal Titration Calorimetry (ITC). The peptides, prepared in DDM solution, were titrated in a solution of BacA_*ef*_ micelles. The titration of EntC into a BacA_*ef*_ solution exhibited an exothermic binding curve, best fitted with a single binding site model and providing a *K*_d_ of 196 nM (Fig. 4*E* and Table 1). The binding was governed by an important enthalpy change (ΔH -23.1 kcal/mol) with a strong entropy penalty (-TΔS of 14.1 kcal/mol). As a control, no binding was observed upon titration of EntC into BacA_*ec*_ (Fig. S7). The titration of EntC1 into BacA_*ef*_ also exhibited an exothermic reaction, providing a *K*_d_ of 306 nM, with a similar free energy change (ΔG -9 kcal/mol) but a much smaller magnitude of heat release (ΔH = −5.4 kcal/mol) and a favorable entropy change (-TΔS -3.4 kcal/mol) (Fig. 4*E* and Table 1). No binding was observed upon titration of EntC2 with BacA_*ef*_, nor titration of EntC2 with EntC1 (Fig. S7). These data demonstrate a favorable binding of EntC1 to BacA_*ef*_, which is consistent with the inhibition of BacA activity and FIDA analyses. It also suggests that EntC2 binds to the BacA_*ef*_:EntC1 pre-complex as the thermodynamic parameters significantly differ when EntC2 was added together with EntC1, while it does not apparently bind directly to BacA_*ef*_, nor to EntC1. Indeed, the titration of EntC2 into a mixture of BacA_*ef*_ and EntC1 at equimolar concentration displayed an exothermic binding curve featured by a favorable enthalpy change (ΔH -16.8 kcal/mol) together with an entropy penalty (-TΔS of 6.5 kcal/mol) (Fig. 4*E* and Table 1). Specifically, the formation of a BacA_*ef*_:EntC1 pre-complex enables the subsequent binding of EntC2. The affinity of this second binding event is high, about 10-fold higher (21 nM) than EntC1 binding, and the stoichiometry was very close to 1:1. Consistently, the titration of EntC1 into a mixture containing BacA_*ef*_ and EntC2 displayed a similar binding pattern as the one obtained with the titration of EntC into BacA_*ef*_ (Fig. S7).

**Table 1:** ITC-determined thermodynamic parameters

Cell	Syringe	*n*	*K*_D_ (nM)	ΔH (kcal.mol^-1^)	-TΔS (kcal.mol^-1^)	ΔG (kcal.mol^-1^)
[BacA_*ef*_]	+ EntC	0.90 ± 0.01	196 ± 47	−23.1	14.1	−9.0
[BacA_*ef*_]	+ EntC1	0.60 ± 0.02	306 ± 127	−5.4	−3.4	−9.0
[BacA_*ef*_]	+ EntC2	No binding				
[BacA_*ef*_/EntC1]	+ EntC2	0.95 ± 0.01	21 ± 8	−16.8	6.5	−10.3
[BacA_*ef*_/EntC2]	+ EntC1	0.69 ± 0.01	480 ± 140	−27.6	19.2	−8.48
[BacA_*ef*_ E20A]	+ EntC	0.69 ± 0.01	190 ± 40	−22.0	13.0	−9
[BacA_*ef*_ S26A]	+ EntC	0.80 ± 0.02	439 ± 125	−17.0	8.5	−8.5
[BacA_*ef*_ R172A]	+ EntC	0.30 ± 0.03	1610 ± 650	−11.4	3.6	−7.8
[BacA_*ef*_ K209A]	+ EntC	0.46 ± 0.02	1837 ± 335	−8.3	0.1	−8.1
[BacA_*ef*_ K209A D110A D106A]	+ EntC	No binding				
[BacA_*ef*_]	+ LcnG	0.62 ± 0.01	165 ± 19	−20.4	11.3	−9.1
[BacA_*ef*_]	+ LcnG_α (LcnG___α___)_	0.58 ± 0.02	898 ± 254	−8.9	0.8	−8.1
[BacA_*ef*_/LcnG_α_]	+ LcnG_β_	0.55 ± 0.01	106 ± 15	−16.8	7.4	−9.4
[BacA_*ef*_/EntC1]	+ LcnG_β_	0.66 ± 0.01	728 ± 134	−21.7	13.5	−8.2
[BacA_*ef*_/LcnG_α_]	+ EntC2	0.77 ± 0.01	898 ± 254	−19.5	10.5	−9.0
[BacA_*ef*_]	+ EntC1 + LcnG_β_	0.79 ± 0.01	307 ± 58	−19.4	10.6	−8.7
[BacA_*ef*_]	+ LcnG_α_ + EntC2	0.55 ± 0.01	220 ± 32	−24.5	15.6	−8.9

ITC thermogram responses for the titration of the EntC or LcnG peptides (400 μM) in the syringe into the specified solution containing the protein BacA_*ef*_ or variants (33 μM) in the measurement cell.

The corresponding thermogram response for each measurement can be found in the main figures or supporting information. The data shown here are representative of experiments performed at least in triplicate.

### BacA_*ef*_ is gradually and highly stabilized upon binding with EntC peptides

ITC measurements strongly suggest that EntC binding stabilizes BacA_*ef*_ according to the enthalpy-driven binding associated with a strong entropy penalty. To further investigate the extent of this stabilization, we measured the melting temperature (T_m_) and enthalpy of denaturation (ΔH) of BacA_*ef*_ in the absence and presence of peptides using Differential Scanning Calorimetry (DSC). Importantly, none of the peptides themselves (individually or combined) displayed a DSC signal when prepared in DDM solution at 66 μM. The thermogram of BacA_*ef*_ at 33 μM displayed a Tm of 66.3°C and a ΔH of 26.7 kcal/mol (Fig. 4*F*). The addition of EntC2 at twice the concentration of BacA_*ef*_ did not change the DSC signal, which correlates with the lack of interaction as already emphasized. In contrast, the thermogram of BacA_*ef*_ in the presence of EntC1 displayed a single endothermal peak with a significant increase of the T_m_ and ΔH to 73°C and 45.3 kcal/mol, respectively (Fig. 4*F*). The addition of complete EntC further enhanced the stabilization of the complex yielding a T_m_ of 83.8 °C and a ΔH of 83.7 kcal/mol (Fig. 4*F*). It is not possible to evaluate the contribution of peptide structuring to these measurements, particularly regarding the ΔH value, as it reflects the overall bonding energy of all components within the complex. Nevertheless, the data unambiguously show that BacA_*ef*_ becomes substantially more stable upon bacteriocin binding, with stability increasing gradually in response to the sequential binding of EntC peptides.

### AlphaFold2 models a reliable BacA_*ef*_:EntC complex

We used AlphaFold2 multimer (26) to predict the binding mode of EntC to BacA_*ef*_. First, a model of BacA_*ef*_ was generated with high confidence (Fig. S8, *A*–*C*): the predicted template modeling (pTm) score was 0.92, and the predicted Local Distance Difference Test (pLDDT) score was 95.52. The BacA_*ef*_ model shares high similarity with the BacA_*ec*_ crystallographic structure, displaying an RMSD of 1.35 Å over all C_α_ atoms (Fig. S8*D*). According to this model, BacA_*ef*_ consists of ten membrane-embedded α-helices, six of which are transmembrane (H3-5, H8-10), while shorter helices H1 and H2, as well as H6 and H7, form two reentrant helix-loop-helix motifs arranged in an inverted manner with respect to the membrane plane. The two short loops from these motifs come in close contact at the middle of the protein, where they form the bottom of a cavity that is open to the outer side of the membrane as well as towards the hydrophobic core of the lipid bilayer between TM helices H4 and H8, forming a V-shape entrance, which is lined by hydrophobic residues. Previous studies of BacA_*ec*_ have identified E21, S27, and R174 as the catalytic residues, which, together with other conserved residues, are located within the helix-loop-helix motifs, at the bottom of the cavity (13, 27). Sequence alignment and structure superposition identified the corresponding catalytic residues in BacA_*ef*_ as E20, S26, and R172 (Fig. S3).

**Figure 5:**
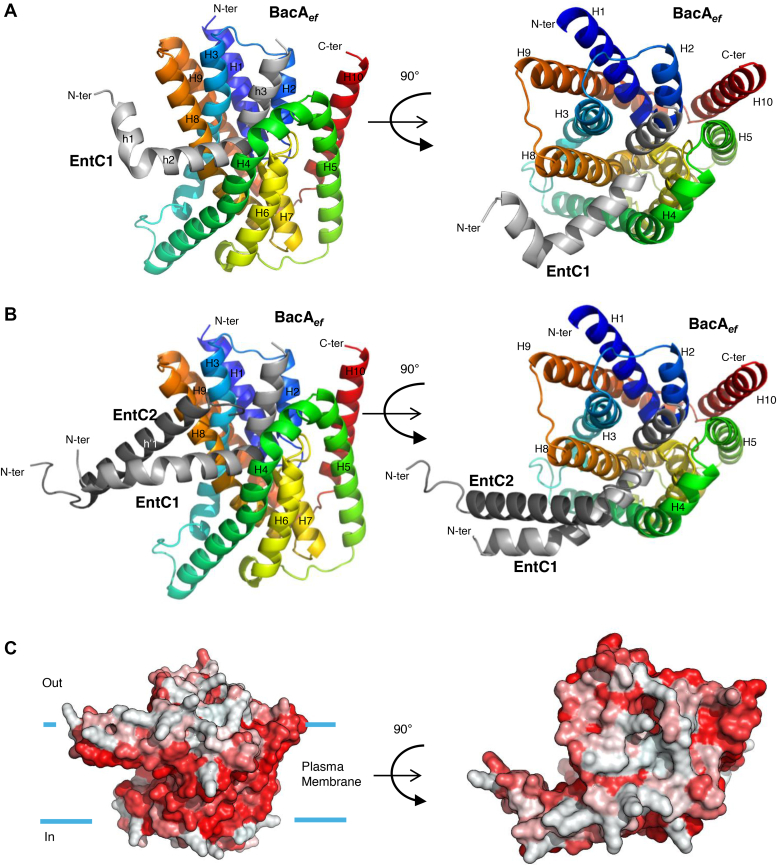
**AlphaFold2 modelling of BacA_*ef*_ in complex with EntC peptides.***A* and *B*, cartoon representation of the best structural model of BacA_*ef*_ in complex with EntC1 (*A*) and with EntC (*B*). BacA_*ef*_ is colored in rainbow from the N- (*blue*) to the C-terminal (*red*), EntC1 is colored in *light gray*, EntC2 is colored in *dark gray*. *C*, surface representation of the BacA_*ef*_:EntC complex with hydrophobic residues according to the Eisenberg hydrophobicity scale colored in *red*. The likely membrane boundaries are indicated by *blue bars*.

**Figure 6:**
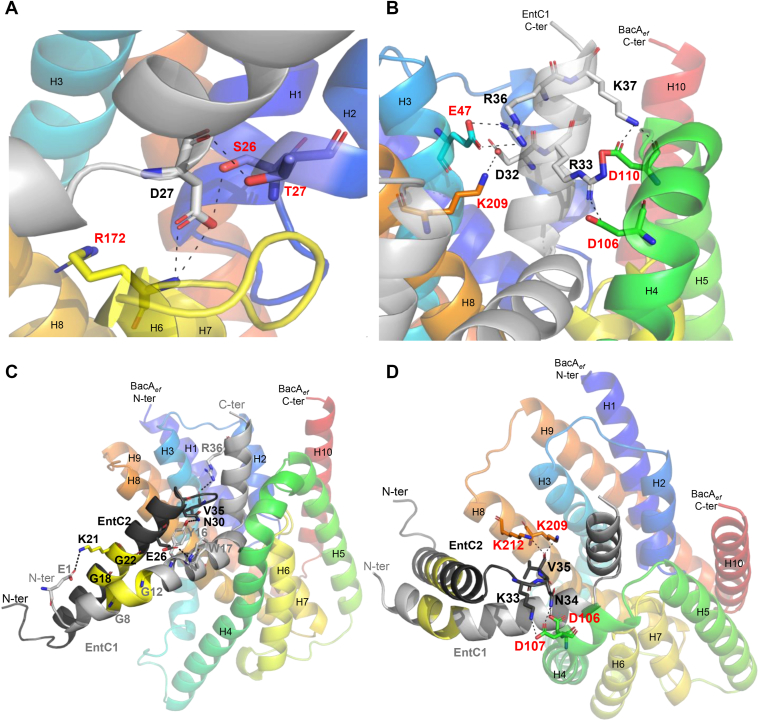
**Details on interfacial regions within AlphaFold2 models of BacA_*ef*_ in complex with EntC peptides.***A* and *B*, zooms at the interface region between BacA_*ef*_ and EntC1. *A*, zoom at the interface region nearby the catalytic site of BacA. *B*, zoom at the interface region at the upper-side entrance of BacA catalytic pocket. *C* and *D*, details of the interface regions formed between EntC2 and EntC1 (*C*) or BacAef (*D*). Residues potentially involved in intermolecular bonds (*dashed lines*), according to PISA analysis, are represented in stick and colored according to the atom (N in *blue*, O in *red*).

The structures of EntC peptides were not confidently predicted, either individually or combined (Fig. S9). In contrast, AlphaFold2 has confidently predicted a BacA_*ef*_:EntC1 complex (Figs. 5*A* and S10, *A* and *B*) with an average pLDDT of 91.06, a pTM score of 0.89, and an ipTM (interface pTM score) score of 0.82. The confidence was only reduced for the N-terminal part of EntC1, which was not predicted to be in contact with BacA. According to this model, EntC1 consists of three short α-helices h1 V_3_-V_11_, h2 G_12_-M_25_ and h3 D_27_-K_38_ (Fig. 5*A*). The h2 and h3 helices are docking deeply within the outward-open cavity of BacA. The helix h2 is passing through the V-shaped entrance, establishing hydrophobic contacts with TM helices H4 and H8, where they are both kinked at invariant proline residues. The helix h1 and a portion of h2 then protrude out of the BacA cavity at the midplane of the membrane (Fig. 5*A*). The h2 and h3 helices form a right angle close to the BacA catalytic site, where EntC1-D27 may form bonds with R172 and S26 catalytic residues of BacA according to PISA analysis of BacA_*ef*_:EntC1 model (Fig. 6*A* and Table S2). Other charged residues from the C-terminus of EntC1 may also form contacts with BacA residues from TM H3 (E47), H4 (D106 and D110) and H8 (K209), which are exposed at the upper side of the cavity entrance (Fig. 6*B* and Table S2).

AlphaFold2 did not predict a complex between BacA_*ef*_ and EntC2. In contrast, a complex between BacA_*ef*_ and both EntC peptides simultaneously was modeled with a high pLDDT score of 94.44, a pTm score of 0.91, and an ipTM score of 0.89 (Figs. 5, *B* and *C* and S10*C*). In contrast to BacA_*ef*_:EntC1 model, the N-terminal part of EntC1 (helices h1 and h2), which is depicted to interact with the cognate EntC2 peptide, was modelled as an extended α-helix with a much greater confidence throughout the helix (pLDDT > 90). The C-terminal part of EntC1 remains in the same position within BacA cavity upon EntC2 binding. EntC2 forms a single α-helix (h1′ P_7_-N_30_) and only its non-structured N-terminal part, Gly1 to Trp5, displayed a lower confidence. There is no major structural difference between BacA_*ef*_ in complex with EntC1 or both peptides (RMSD = 0.138 Å). According to PISA analysis of BacA_*ef*_:EntC model, the C-terminal K33, N34 and V35 residues of EntC2 may contact BacA residues located at the outer ends of TM H4 (D106 and D107) and H8 (K209 and K212). Hydrophobic contacts may also occur between EntC2 (A20, I23 and W32) with residues lining the hydrophobic groove formed by TM helices H4 and H8 from BacA_*ef*_ (Fig. 6, *C* and *D* and Table S2). A GxxxG motif is conserved in both peptides of all known class IIb bacteriocins (28, 29) (Fig. 1*A*). These motifs were demonstrated to be essential for the antibacterial activity of the bacteriocins, where they were hypothesized to drive tight peptide-peptide interaction (30, 31). In line with this, the G_8_ x x x G_12_ motif from EntC1 is in very close contact with the G_18_ x x x G_22_ motif from EntC2 in BacA_*ef*_:EntC model (Fig. 6, *C* and *D*). The N-terminal to mid-region of EntC peptides, featured by this tight helix-helix contact, are likely deeply embedded in the membrane hydrophobic acyl core, which is relevant with their amphipathic nature as emphasized by helical wheel projections and the calculation of their hydrophobic moment by Heliquest software (32) (Fig. S11). According to the model, EntC binding to BacA induced only a minor change in the positioning of the BacA α-helices (Fig. S12) (RMSD = 0.538 Å). The most notable change is a small shift of the ends of TM α-helices that are exposed to the outer side of the membrane leading to a small dilation of the aperture, but the protein cavity remains sealed towards the cytoplasmic side of the membrane.

### Specific BacA_*ef*_ residues determine EntC binding

**Figure 7:**
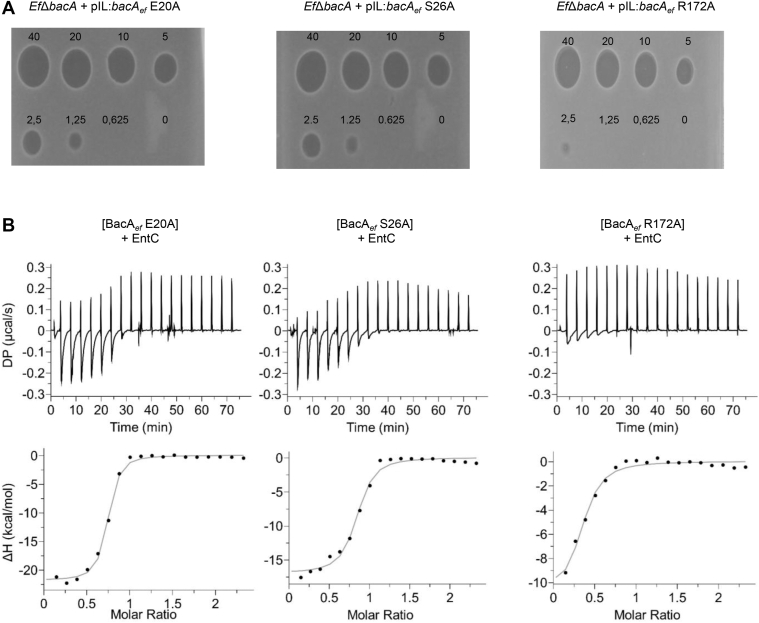
**Effect of substitution of BacA_*ef*_ catalytic residues on the activity of EntC.***A*, spot-on-lawn assays of EntC on a lawn of *E. faecalis Ef*Δ*bacA* cells expressing catalytically inactive variants of BacA_*ef*_. *B*, ITC scans of BacA_*ef*_ variants titration with EntC. *Upper panels*, raw ITC thermograms from the titration of BacA_*ef*_ variants (33 μM) with EntC bacteriocin (400 μM). *Lower panels*, heat profile from peak integration of ITC thermograms to determine the thermodynamic parameters of the interaction. The data shown are representative of experiments performed in triplicate.

To assess the requirement for an active BacA_*ef*_ for EntC activity and to highlight key residues involved in the formation of a complex, we generated BacA_*ef*_ catalytic inactive variants through alanine substitution of E20, S26 and R172 residues. The variants were assessed by a functional complementation assay using the *E. coli* BWTetra-Ts*bacA* strain, which lacks all chromosomal genes encoding C_55_-PP phosphatase and carries a copy of *bacA*_*ec*_ on a thermosensitive plasmid (27). This strain grows normally at 30 °C but lyses at 42 °C due to the arrest of C_55_-P recycling unless it is complemented *in trans*. The growth of this strain was restored at 42 °C upon the expression of *bacA*_*ef*_ from a plasmid, while the mutation of any catalytic residue invalidated the complementation (Table S1). The susceptibility of the *E. faecalis Ef*Δ*bacA* strain to EntC was restored with a plasmid expressing wild-type *bacA*_*ef*_ or any of the three catalytic site mutant alleles, although a slight decrease of the susceptibility was observed with R172A variant as judged from spot-on-lawn assays (Fig. 7*A*). In conclusion, BacA functionality is not required for EntC antibacterial activity and although S26 and R172 are, according to AlphaFold2, involved in the contact, their mutation do not abolish BacA targeting. We also measured the bactericidal effect of EntC on *Ef*Δ*bacA* cells expressing the latter variants. When applied for 30 min on exponentially growing cells, 0.5 μM of EntC was found to kill more than 99% of the cells expressing wild-type BacA_*ef*_ (Table 2). The expression of the variant E20A did not significantly change the susceptibility to EntC as compared to the wild-type, while the expression of S26A and R172A variants significantly increased the viability, up to 6.8% and 7.8%, respectively (Table 2). The BacA_*ef*_ variants were purified to further assess EntC binding. As anticipated, these variants were almost completely lacking any C_55_-PP phosphatase activity (Table S1). Titrations of EntC to BacA_*ef*_E20A displayed a very similar binding pattern as that determined with wild-type BacA_*ef*_ (Table 1 and Fig. 7*B*). In contrast, in line with the AlphaFold model of BacA_*ef*_:EntC complex suggesting the involvement of S26 and R172 residues in the interaction, the titration of S26A and R172A BacA_*ef*_ variants with EntC showed a less favorable interaction as compared to the wild-type, displaying an increase of the apparent *K*_d_ (439 ± 125 nM and 1610 ± 650 nM, respectively), a decrease in the heat release and a reduction in the entropy penalty upon binding (Table 1 and Fig. 7*B*).

**Table 2:** Survival rates of *E. faecalis Ef*Δ*bacA* cells expressing BacA_*ef*_ variants from pIL:*bacA*_*ef*_ plasmids after 30 min of exposure with 0.5 μM EntC

% of survival^a^	Control	+ 0.5 μM EntC^b^
Protein		
BacA_*ef*_	100 ± 7	<1
BacA_*ef*_ E20A	100 ± 11	4.0 ± 3.7
BacA_*ef*_ S26A	100 ± 7	6.8 ± 2.4^c^
BacA_*ef*_ R172A	100 ± 16	7.8 ± 0.8^d^
BacA_*ef*_ D106A	100 ± 5	1.6 ± 1.0
BacA_*ef*_ D110A	100 ± 3	2.8 ± 1.0
BacA_*ef*_ K209A	100 ± 10	11 ± 1.0^d^
BacA_*ef*_ K209A D106A	100 ± 22	14 ± 4.0^c^
BacA_*ef*_ K209A D110A	100 ± 9	7.6 ± 1.0^e^
BacA_*ef*_ D106A D110A	100 ± 19	4.0 ± 3.0
BacA_*ef*_ K209A D106A D110A	100 ± 14	54 ± 11^d^

a The survival rates are expressed as a percentage of survival rates obtained with the same cells without EntC exposure. The survival rate represents the average number of CFU obtained from three independent experiments, numerated after 30 min of incubation in the presence or not of EntC added at 0.5 μM final concentration.

b The *p* values were calculated for each variant in comparison with wild-type BacA_*ef.*_

c *p* ≤ 0.01.

d *p* ≤ 0.0001.

e *p* ≤ 0.001.

**Figure 8:**
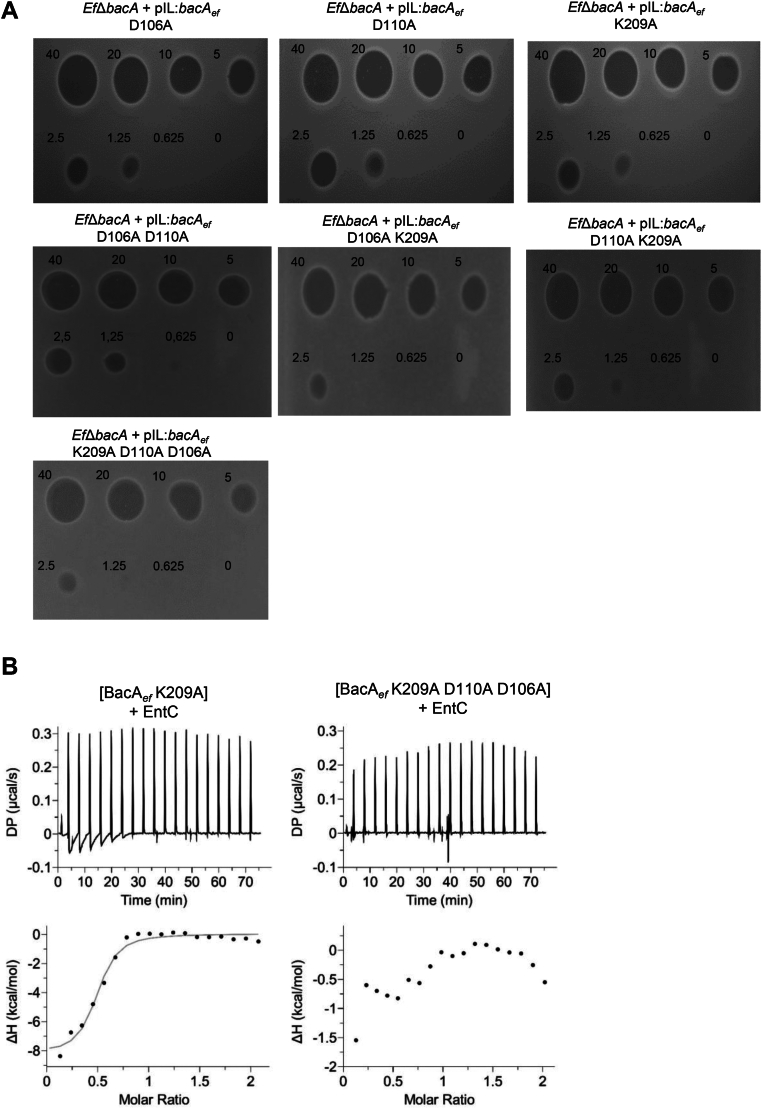
**Effect of substitution of specific BacA_*ef*_ residues on the activity of EntC.***A*, Spot-on-lawn assays of EntC on a lawn of *E. faecalis Ef*Δ*bacA* cells expressing BacA_*ef*_ variants. *B*, ITC scans of BacA_*ef*_ variants titration with EntC. *Upper panels*, raw ITC thermograms from the titration of BacA_*ef*_ variants (33 μM) with EntC bacteriocin (400 μM); *Lower panels*, heat profile from peak integration of the ITC thermograms to determine the thermodynamic parameters of the interaction. The data shown are representative of experiments performed in triplicate.

We also performed alanine substitution of D106, D110, and K209 residues of BacA_*ef*_ since they may also be involved in contacts with both EntC1 and EntC2 according to our predictions (Fig. 6, *B* and *D* and Table S2). In contrast to the catalytic mutants, all the variants tested (combinations of single, double and triple mutations) complemented the *E. coli* BWTetra-Ts*bacA* strain, demonstrating the functionality of these variants (Table S2). Upon their expression in *Ef*Δ*bacA* strain, only the K209A variants caused a small reduction in susceptibility when compared to the wild-type BacA_*ef*_ as judged from spot-on-lawn assays (Fig. 8*A*). When we measured the bactericidal effect of EntC on BacA_*ef*_ variants-expressing cells, the K209A mutation resulted in a significant gain of survival rate, up to 11% as compared to the wild-type, while the D106A and D110A mutations did not (Table 2). The double mutants (K209A-D106A and K209A-D110A) did not significantly increase survival rates as compared to K209A variant, while the triple mutant (K209A-D106A-D110A) displayed 54% of survival rate (Table 2). The purified BacA_*ef*_K209A and BacA_*ef*_ triple mutants displayed 10% of residual C_55_-PP phosphatase activity as compared to wild-type BacA_*ef*_ (Table S1), demonstrating a significant role of K209 residue in BacA_*ef*_ activity, although this residual activity was sufficient for *in vivo* complementation. EntC titration on BacA_*ef*_K209A revealed an impaired interaction, as compared to the wild-type BacA_*ef*_, characterized by a higher *K*_d_ (1837 ± 335 nM) and a reduction of heat content release, and no binding was observed upon titration of EntC on the triple mutant (Fig. 8*B* and Table 1). Since the lack of interaction could originate from an impaired stability of the latter variant, its thermal stability was compared to that of wild-type BacA_*ef*_ (Fig. S13). The higher Tm and similar ΔH values of the variant as compared to the wild-type indicated that the lack of interaction cannot be assigned to impaired stability.

### Cross-reactivity of EntC-related bacteriocins

**Figure 9:**
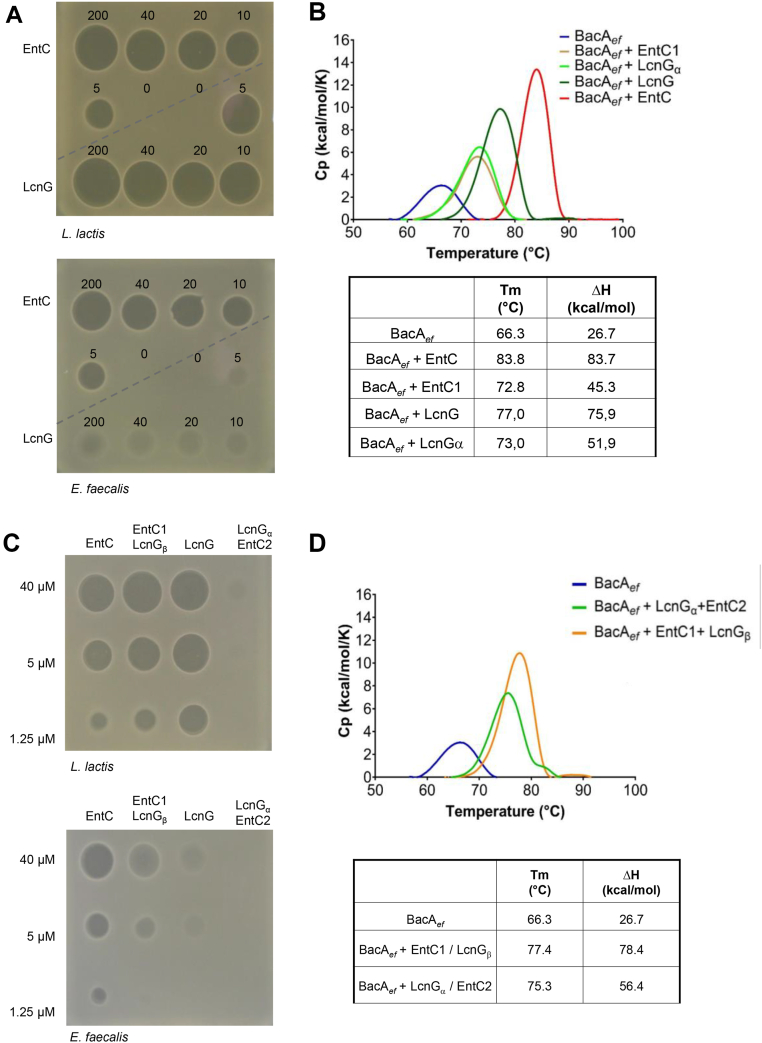
**Cross-activity of EntC and LcnG peptides and chimeric bacteriocins.***A*, activity measurement of EntC and LcnG peptides towards *L. lactis* and *E. faecalis* cells. *B*, stability measurement of BacA_*ef*_ in the absence or in the presence of different peptide solutions. *Upper panel*, representative DSC thermograms of BacA_*ef*_ (33 μM) incubated with different peptide mixtures (66 μM), *lower table*, T_m_ and thermodynamic parameters. *C*, activity measurement of EntC and LcnG chimera on *L. lactis* (*upper panel*) and *E. faecalis* (*lower panel*) strains. The data shown are representative of experiments performed in triplicate. *D*, stability measurement of BacA_*ef*_ in the absence or in the presence of bacteriocin chimera. *Upper panel*, representative DSC thermograms of BacA_*ef*_ (33 μM) incubated with chimera (66 μM), *lower table*, Tm and thermodynamic parameters.

To date, only a limited number of EntC-related bacteriocins have been identified, they are categorized as Enterocins and Lactococcins based on the producing cells (Fig. 1*A*). To investigate their specificity of action, we examined the cross-activity of LcnG and EntC peptides. Chemically synthesized LcnG peptides (61% identity with EntC) were obtained. *L. lactis* IL1403 exhibited sensitivity to both bacteriocins, while *E. faecalis* was only susceptible to EntC (Fig. 9*A*). Alignment of BacA from these two species revealed conservation of residues involved in BacA_*ef*_:EntC interaction according to AlphaFold (Fig. S3 and Table S2). *In vitro*, LcnG inhibited BacA_*ef*_ C_55_-PP phosphatase activity in a dose-dependent manner similar to EntC (IC_50_ value of LcnG measured at 0.8 μM) (Fig. S14). LcnG_α_ (EntC1 homolog) also inhibited BacA_*ef*_ activity with a comparable IC_50_ value (1 μM), while LcnG_β_ had no effect (Fig. S14). Unfortunately, we were unable to produce a stable form of BacA from *L. lactis* for a comprehensive *in vitro* comparative study.

We further assessed LcnG binding to BacA_*ef*_ by ITC (Table 1 and Fig. S15). LcnG titration on BacA_*ef*_ exhibited an exothermic reaction with thermodynamic parameters similar to EntC titration, characterized by a substantial enthalpy release (−20.4 kcal/mol) and a large entropy penalty (11.3 kcal/mol), and a *K*_d_ of 165 nM. LcnG_α_ titration exhibited a significantly higher *K*_d_ compared to the complete bacteriocin (898 nM). As anticipated, LcnG_β_ titration showed no interaction with BacA_*ef*_. Titration of LcnG_β_ on the BacA_*ef*_ + LcnG_α_ mixture displayed a strong enthalpy-driven interaction (ΔH of −16.8 kcal/mol), with an entropy penalty (-TΔS of 7.4 kcal/mol) and the *K*_d_ was lower (106 nM) as compared to LcnG_α_. Overall, these results demonstrate a binding pattern of LcnG to BacA_*ef*_ similar to EntC, although LcnG was found to be inactive against *E. faecalis*.

We further assessed the structural similarity of the complexes formed by BacA_*ef*_ with both bacteriocins by DSC (Fig. 9*B*). The addition of LcnG_α_ to BacA_*ef*_ resulted in an increase in BacA T_m_ and ΔH in a manner analogous to EntC1. The presence of both LcnG peptides further increased T_m_ and ΔH values, albeit not to the same extent as EntC. Despite LcnG binding to BacA with similar thermodynamic parameters as EntC, thermal stability measurements revealed significant differences between the complexes they form, with differences of 6.8 °C (T_m_) and 7.8 kcal/mol (ΔH). AlphaFold2 generated a high-confidence model of BacA_*ef*_ in complex with LcnG, exhibiting an average pLDDT of 95.04, a pTM score of 0.92, and an ipTM of 0.9 (Fig. S16). This model indicates that LcnG is positioned in a nearly identical manner as to EntC in the BacA_*ef*_ outward-open cavity, with an RMSD of 0.2 Å. A noticeable difference between BacA_*ef*_:LcnG and BacA_*ef*_:EntC complexes may account for distinct activities. This may be attributable to the lack of a putative ionic bond between LcnG_α_ and K209 residue from BacA_*ef*_, resulting from the lack of an acidic residue in LcnG_α_ as compared to D32 from EntC1 (Figs. 6*B* and S16*B*).

### Activity of chimera bacteriocins

To elucidate the determinants for the specificity of these bacteriocins, chimera bacteriocins were evaluated for their antibacterial activity and their binding to BacA_*ef*_ *in vitro*. The LcnG_α_-EntC2 chimera (LcnG_α_ and EntC2 in 1:1 M ratio) was inactive against both *E. faecalis* and *L. lactis* (Fig. 9*C*). Previous mutational studies have shown that residue E1 in Ent1071A was essential for the bacteriocin's activity (Ent1071 is different from EntC by only one residue between Ent1071B and EntC2, Fig. 1*A*). In line with this, when LcnG_α_ was paired with EntC1071B, it was shown to be inactive due to the lack of a negatively charged residue at position 1 in LcnG_α_ (29). Therefore, the lack of activity of LcnG_α_-EntC2 combination may be similarly assigned the lack of an acidic residue in position 1 in LcnG_α_. Interestingly, EntC1 E1 residue possibly forms a salt bridge with EntC2 K21 residue according to PISA analysis of BacA_*ef*_:EntC complex (Fig. 6*C*). Conversely, the EntC1-LcnG_β_ chimera exhibited a similar antibacterial activity to EntC and LcnG against *L. lactis*, and it also displayed a moderate activity against *E. faecalis* (Fig. 9*C*).

We further analyzed the binding of the chimera to BacA_*ef*_ (Table 1 and Fig. S17). The titration of LcnG_α_-EntC2 chimera on BacA_*ef*_, or titration of EntC2 to a BacA_*ef*_ + LcnG_α_ mixture, both exhibited exothermic binding curves, displaying similar *K*_d_ and thermodynamic parameters to native bacteriocins. Similarly, the titration of EntC1-LcnG_β_ on BacA_*ef*_, or the titration of LcnG_β_ to a BacA_*ef*_ + EntC1 mixture, demonstrated exothermic binding curves. These findings show that binding of the peptides to BacA is not the sole critical determinant for their activity since despite similar binding patterns to BacA_*ef*_, the bacteriocins or chimera display distinct antibacterial activities.

Finally, we analyzed the resulting complexes formed between the chimera and BacA_*ef*_ by DSC (Fig. 9*D*). BacA_*ef*_ was stabilized through the binding of LcnG_α_-EntC2 chimera to a similar extent as LcnG_α_ binding, while the EntC1-LcnG_β_ chimera had a more pronounced effect that is comparable to LcnG’s impact. These results infer distinct conformational states or dynamics induced by the different combinations. EntC1 and LcnG_α_ exhibited the least effect, while EntC caused the most significant stabilization, which correlates with their toxic activity against *E. faecalis*.

## Discussion

In the present work, we have demonstrated that EntC specifically targets the C_55_-P recycling protein BacA through a direct and high-affinity binding. This binding event elicits the dissipation of proton gradient as observed when EntC was added to BacA_*ef*_-containing liposomes, supporting the hypothesis that the membrane permeabilization is the primary cause for the death of the target cells. It further demonstrates that BacA is required and sufficient for EntC to trigger membrane disruption. AlphaFold modelling and interaction assays demonstrate that EntC1 initiates the binding by docking to the outward-open cavity of BacA, establishing intermolecular bonds with deeper BacA catalytic residues. This binding mode of EntC1 to BacA likely mimics the mode of access of C_55_-PP substrate from the outer leaflet of the lipid bilayer. The negatively charged headgroup of C_55_-PP must interact with the active site residues, especially with positively charged R174, localized at the bottom of the catalytic pocket, which corresponds to the midplane of the membrane. The C_55_-PP lipid tail should then exit the cavity along the hydrophobic groove shaped by TM helices H4 and H8 and protrude dynamically within the lipid bilayer. Although a complex of BacA with its substrate has not yet been resolved, this mode of substrate binding was strongly supported by the presence of monoolein lipid, presumably mimicking C_55_-PP, within the active site pocket in the crystal structure of BacA_*ec*_ (14). According to its binding site, EntC1 binding must then block substrate access and thus inhibit BacA catalysis as observed experimentally. ITC measurements of EntC1 binding to BacA reveal an entropy-driven binding event, suggesting the release of ordered water molecules from the binding interface and/or the maintenance of conformational flexibility within the bipartite complex. This aligns with the modeled position of EntC1 within BacA's cavity, where the binding of EntC1 to the hydrophobic pass leading to the cavity would promote the release of ordered water molecules, contributing to the overall increase of the entropy (33, 34, 35). Moreover, according to our model, the N-terminal to mid-region of EntC1 is protruding out of BacA within the hydrophobic core of the lipid bilayer, likely conferring a high degree of freedom to this region (34).

As demonstrated here, the binding of EntC1 enables the subsequent docking of EntC2 to BacA. This observation is consistent with AlphaFold2 modeling, which indicates that EntC2 interacts peripherally in the tripartite complex, establishing contacts with the protruding N-terminal to mid-region of EntC1 as well as with residues from BacA positioned at the ends of TM helices forming the V-shape opening towards the catalytic pocket. This second binding event is characterized by a substantial heat release and a significant entropy penalty. The close helix-helix contacts between the two peptides may significantly reduce the flexibility of EntC1, potentially accounting for the entropy cost and for the strong stabilization of the tripartite complex.

According to the model, the binding of EntC peptides with BacA involves their respective mid-region to C-terminus (residues 20–39 in EntC1 and 21–35 in EntC2), which exhibit high sequence similarity between the related bacteriocins (c.a. 80% identity), while their N-terminal halves are more divergent (c.a. 40% identity) (Fig. 1*A*). This observation aligns with the fact that EntC and LcnG peptides, as well as any chimeric combinations, were found to interact with BacA_*ef*,_ yielding similar thermodynamic parameters. Nevertheless, EntC is the only bacteriocin displaying antibacterial activity against *E. faecalis,* suggesting that beyond their binding to BacA, the conformation or dynamics of the tripartite complex, particularly the tight helix-helix motif of the bacteriocin that protrudes within the lipid bilayer, is critical. This likely accounts for the lack of consistency between BacA binding and antibacterial activity. The reason for LcnG’s inactivity against *E. faecalis* remains unclear, considering that the docking to BacA_*ef*_ is conserved except for a possible salt bridge between EntC1 acidic residue, D32, and BacA_*ef*_ K209 residue according to AlphaFold predictions. Obviously, the N-terminal halves of the peptides have evolved more rapidly than their C-terminal counterparts, which is consistent with the fact that their evolution is not constrained by binding to the cell surface receptor. Evidently, the docking of the peptides *via* their C-terminal halves does not confer the killing activities *per se*, since similar docking patterns do not necessarily result in antibacterial activities, as exemplified by the lack of activity of LcnG or chimera against *E. faecalis*. Therefore, their protruding halves, whose folding does not strictly depend on BacA, may be pivotal for the killing activity.

The cognate peptides interact primarily *via* their respective GxxxG sequences, which have been previously identified as essential for the antibacterial activity of all class IIb bacteriocins (5), despite the fact that these bacteriocins do not apparently share a common cell surface receptor. This further supports the hypothesis that this common helix-helix motif of class IIb bacteriocins is not directly involved in the recognition of the receptor but rather interacts with the membrane to facilitate permeabilization. In EntC/LcnG bacteriocins, this tight helix-helix motif protrudes deeply in the lipid bilayer due to the docking of the C-terminal parts of the peptides to the outward-open cavity of BacA, passing *via* the V-shaped entrance leading to the acyl core of the membrane. The docking of the peptides to BacA likely provides the driving force required for the peptides to insert deeply within the lipid bilayer and cause membrane permeabilization. These GxxxG motifs constitute a prevalent transmembrane helical dimerization interface in which the C_α_-H from two glycine residues from one helix form hydrogen bonds with the carbonyl from the nearby helix, enabling very close contact of the membrane-embedded helices with minimal steric hindrance (36). Recent studies have highlighted the role of GxxxG motifs in the toxicity and pore formation activity of amyloid peptides through the strong stabilization of ion-channel-like pores (37). Notably, the docking of EntC provides substantial stabilization of the tripartite complex and the degree of stabilization was found to correlate with the level of antibacterial activity when comparing the effect of native and chimera bacteriocins. The conformation of a tripartite complex with the least degree of freedom in the membrane apparently provides the optimal support for membrane permeabilization. As previously emphasized, a negative charge at position 1 in EntC1071/EntC1 was shown to be essential for antibacterial activity, and the lack of equivalent residue in LcnG_α_ accounted for the lack of activity when paired with Ent1071B/EntC2 (29). This observation aligns with the lack of antibacterial activity of LcnG_α_-EntC2 chimera. Interestingly, the detrimental effect of removing E1 from EntC1071A is neutralized by introducing another negative charge at position 10 in Ent1071B, restoring antibacterial activity (29). According to our model, E1 from EntC1 may form an ionic bond with K21 from EntC2, and position 10 in EntC2 is in very close proximity to E1 (Fig. 6*C*), suggesting that introducing a negative charge at this position could establish the same ionic pairing with K21, thereby compensating the removal of E1. Since this region of the peptides is expected to reside in the acyl core of the bilayer, we can hypothesize that this ionic bond is essential to maintain and stabilize the peptides anchored to the bilayer through the neutralization of charged residues. Overall, these observations support a key role of the N-terminal halves of the peptides in membrane permeabilization.

Although the peptide EntC1 directly interacts with BacA_*ef*_ and enables the subsequent binding of EntC2, several pieces of evidence suggest that both peptides may initially interact when they come into contact with the membrane, prior to their docking to BacA. This interaction is supported by the lower *K*_d_ value measured when titrating EntC to BacA_*ef*_ as compared to EntC1, as well as the lower IC_50_ value measured for the inhibition of BacA_*ef*_ activity by EntC as compared to EntC1. Furthermore, this reasoning aligns with previous spectroscopy studies of LcnG peptides, revealing a significant enhancement of their helical content when both peptides were combined together with membrane-like environments, suggesting that cognate peptides interact to form one functional unit before docking to their cell surface receptor (4, 24, 25). The interaction of the peptides at the contact of the membrane would enable a single binding event with BacA through lateral diffusion at the membrane surface rather than relying on diffusion in the aqueous environment and sequential interactions of the peptides with their receptor (38).

Our findings indicate that the inhibition of BacA activity is not the primary cause of toxicity, since BacA is not essential in *E. faecalis* as demonstrated here, likely due to its redundancy with other C_55_-PP phosphatases and C_55_-P flippases. Instead, our findings strongly suggest that the lethal activity of these bacteriocins is attributable to membrane permeabilization. This is consistent with the high potency of these bacteriocins, as membrane permeabilization depends on a low number of peptides, unlike what is expected when cell death is promoted by the inhibition of a protein activity. Given the amphipathic nature of the peptides and the mode of docking to BacA, EntC likely resides deep within the lipid bilayer but rather in a parallel orientation with respect to the membrane plane, without crossing the entire membrane, contrary to previous hypotheses (4). EntC insertion could subsequently induce a local thinning or deformation of the membrane, resulting in an imbalance of charge, area, and surface tension that may drive the local disruption of the bilayer structure and cause leakage. This mechanism was hypothesized to be the basis for the antibacterial activity of several amphipathic peptides that affect cell membrane integrity, known as interfacial activity model (39, 40). Our study provides novel mechanistic insights into bacteriocin-target interactions and identifies BacA as a valuable target for bacteriocin-mediated killing. These discoveries significantly advance our understanding of class II bacteriocins and open avenues for the rational design of peptide-based antimicrobials with enhanced specificity and potency.

## Experimental procedures

### Products

The oligonucleotides were provided and DNA sequencing was performed by Eurofins Genomics. *n*-dodecyl-β-D-maltopyranoside (DDM) was provided by ThermoScientific. Farnesyl pyrophosphate (C_15_-PP) and isopentenyl pyrophosphate (C_5_-PP) were from Sigma, and [^14^C]C_5_-PP ((1.48–2.22 GBq)/mmol) was from PerkinElmer. DOPC, DOPE and DOPG lipids were provided by Avanti Polar Lipids. C_55_-PP synthase UppS was purified as previously described (10). [^14^C]C_55_-PP was prepared from its precursors, C_15_-PP and [^14^C]C_5_-PP, upon reaction with UppS according to the published procedure (10). DNA purification kits were from Macherey-Nagel. EntC and LcnG peptides were custom-synthesized by GenScript using chemical synthesis, and their purity (>90%) was confirmed by reverse-phase HPLC. All other materials were of reagent grade and obtained from commercial sources. *E. coli* strains were grown in 2xYeast Extract and Tryptone (2YT) medium, at 30, 37 or 42 °C with aeration and agitation (200 rpm). Solid media were obtained by adding 15 g/L agar. *E. faecalis* strains were grown in brain heart infusion (BHI) medium at 30 or 37 °C in static conditions, and when specified in M17 supplemented with 0.5% (w/v) glucose (M17G) or Man–Rogosa–Sharpe (MRS) medium. *L. lactis* strain Il1403 was grown in M17G at 30 °C in static conditions. The antibiotics ampicillin (Amp) and chloramphenicol (Cm) were used at 100 μg/ml and 25 μg/ml, respectively. Erythromycin (Ery) was used at 3 μg/ml when used for *E. faecalis* and 150 μg/ml for *E. coli*.

### Deletion of *bacA*_*ef*_

The *Ef*Δ*bacA* strain was generated by double-crossover recombination in the V583-derivative strain, VE18379 (41), designated as the wild-type strain (WT) in the text and figures for clarity. For that purpose, two DNA fragments encompassing the 5′- and 3′-ends of the *bacA* gene were PCR amplified from WT chromosomal DNA with primer pairs OEF1066-OEF1067 and OEF1068-OEF1069, respectively (Table S3). The fragments were cloned by Gibson assembly into pGhost9 vector (42) at the EcoRV site, yielding the plasmid pGhost:*bacA*_*ef*_. A markerless in-frame *bacA* deletion mutant was selected as previously described (43), and the deletion of *bacA* was confirmed by sequencing of the genomic DNA of the strain.

### Cloning

For complementation experiments of *Ef*Δ*bacA* strain, *bacA*_*ef*_ was cloned in a low copy number *E. coli*-*E. faecalis* shuttle plasmid. The entire gene was amplified with primers OEF1170-OEF1171 (Table S3) and cloned by Gibson assembly into pIL252 at the SmaI site. To facilitate targeted mutagenesis, the p15A replication origin amplified with OEF1145-OEF1146 primers and digested with NheI restriction enzyme was further introduced at the XbaI site. The resulting plasmid pIL:*bacA*_*ef*_ was electroporated into *Ef*Δ*bacA* strain. *Ef*Δ*bacA* strain harboring the empty pIL252 plasmid was used as a control. For recombinant expression of BacA in the *E. coli* C43(*DE3*) strain, the *bacA*_*ef*_ gene was amplified with primers BacA1-BacA2 (Table S3). The resulting fragment was inserted at BamHI and HindIII sites in the pET2130 vector (44) generating pET:*bacA*_*ef*_. Plasmid pTrcBac30 was used for the expression of N-terminal His_6_-tagged BacA_*ec*_ (10), while plasmid pPgpBHis was used for the expression of N-terminal His_6_-tagged PgpB (45). Variants were produced by site-directed mutagenesis using the QuickChange II Site-Directed Mutagenesis Kit (Agilent Technologies) with the original plasmid as a template and a pair of primers containing the mutation (Table S3). The mutations were confirmed by sequencing the entire *bacA* gene. Electrotransformation of *E. coli* and *E. faecalis* was carried out as previously described (46, 47).

### EntC or LcnG agar-assay plate on *E. faecalis* or *L. lactis*

*E. faecalis* and *L. lactis* strains were grown in BHI and M17G media, respectively. The selective antibiotic Ery was added if the strain carries a plasmid. When absorbance at 600 nm (A_600_) reached 0.6, the culture was diluted to 10^6^ CFU/ml in the corresponding media containing 0.75% agar. The mix was spread on an agar plate of the corresponding media, and 6 μl-droplets of EntC or LcnG peptide solutions, at various concentrations, were spotted. The plates were incubated overnight at 30 °C or 37 °C.

### Survival test on *E. faecalis*

A culture at exponential growth (A_600_ ∼0.4, corresponding to about 3 x 10^8^ CFU/ml) of *Ef*Δ*bacA* strain transformed by the pIL:*bacA*_*ef*_ or derivative plasmids was diluted to 10^6^ CFU/ml in BHI in the presence or absence of EntC at 0.5 μM. After 30 min of incubation at 37 °C, serial dilutions were plated on BHI-Ery agar plates and incubated overnight at 37 °C to determine the viable cell counts. The rate of survival was expressed as a percentage of CFU obtained after exposure to EntC as compared with the control without EntC.

### Expression of recombinant proteins

The His_6_-tagged proteins were expressed in the *E. coli* C43(*DE3*) cells from pET:*bacA*_*ef*_ plasmid. The cells were grown in 2YT medium supplemented with ampicillin at 37 °C until A_600_ reaches 1. Protein production was then induced by the addition of 1 mM IPTG and the culture was placed at 22 °C and rotated at 180 rpm, for 16 h. Cells were harvested by centrifugation at 4000*g* for 20 min at 4 °C and washed by resuspension in buffer A (20 mM HEPES, pH 7.5, 0.4 M NaCl, 10% (v/v) glycerol). The bacterial pellet was stored at −20 °C until protein purification.

### Purification of membrane proteins

Cells were resuspended in buffer A and lysed using a French press. The membrane fraction was obtained after ultra-centrifugation at 186,000*g* for 45 min at 4 °C. The membrane pellet was resuspended in 20 ml of buffer B (buffer A + 2% (w/v) DDM) and incubated for 2 h with agitation at 4 °C for solubilization, followed by centrifugation at 165,000*g* for 25 min at 4 °C. One mL of Ni^2+^-nitrilotriacetic acid agarose resin (Qiagen) pre-equilibrated in buffer C (buffer A + 0.1% DDM) was added to the supernatant, and the mixture was incubated overnight at 4 °C under agitation. It was then transferred into a chromatography column Poly-prep (Biorad), allowing the resin coupled with the proteins to settle, and was further washed with buffer C. The column was then washed with 30 ml of buffer C + 10 mM imidazole. Proteins were eluted in 1-ml fractions with buffer C containing 400 mM imidazole. Fractions were quantified by absorbance at 280 nm and analyzed on SDS-PAGE (12% acrylamide). Those containing the target protein were subjected to gel filtration on a Superdex 200 pg column in buffer D (buffer C with NaCl at 0.2 M) at 1 ml/min flow rate. 1 ml-fractions were collected, quantified by absorbance at 280 nm and analyzed by SDS-PAGE coupled Coomassie blue staining or western blotting using an anti-His tag antibody (Penta-His HRP conjugate from Qiagen, RRID: AB_3699318). Protein fractions were concentrated using a Vivaspin 20 concentrator with a 50 kDa cut-off up to 1 mg/ml as determined by the absorbance at 280 nm using extinction coefficients of 44,920 M^−1^ cm^−1^ and 25,440 M^−1^ cm^−1^ for BacA_*ef*_ and BacA_*ec*_, respectively. Samples were stored at −20 °C. ITC and DSC measurements were carried out with a BacA sample at 1 mg/ml dialyzed overnight against the following buffer: 20 mM Tris-HCl, pH 7.5, 0.2 M NaCl and 0.1% DDM.

### C_55_-PP phosphatase assay in detergent

The C_55_-PP phosphatase assays were performed in a 10-μl reaction mixture containing 20 mM Tris-HCl, pH 7.4, 400 μM CaCl_2_, 150 mM NaCl, 0.1% (w/v) DDM and 50 μM radiolabeled [^14^C]C_55_-PP (900 Bq). The BacA concentration was adjusted in order to achieve less than 30% substrate hydrolysis. The mixture was incubated for 10 min at 37 °C, and the reaction was stopped by freezing in liquid nitrogen. Substrates and products were separated by thin-layer chromatography on precoated silica gel 60 plates (Merck) using 1-propanol/ammonium hydroxide/water (6:3:1, v/v/v) as the mobile phase. Radioactive spots were located and quantified using a radioactivity scanner (model Multi-Tracermaster LB285, Berthold-France). When the phosphatase activity was tested at different pH values, the following buffers were used at 20 mM: sodium acetate (pH 4–7) or Tris-HCl (pH 7–8). In the EDTA assay, the protein was pre-treated with 200 μM EDTA. For pH and EDTA assays, CaCl_2_ was omitted from the reaction buffer.

### BacA-containing proteoliposomes preparation

The reconstitution of BacA and PgpB in liposomes was achieved through adapted protocols previously described (48). Liposomes were prepared by rehydrating a lipid film of DOPC: DOPG: DOPE (3 mg: 0.75 mg: 1.25 mg) after 4 h of desiccation by adding 1 ml of buffer (20 mM HEPES, pH 7.5, 50 mM NaCl, 15% glycerol). For the proteoliposomes that were further used for membrane permeabilization assays, the reconstitution was performed in 20 mM HEPES buffer at pH 8 and in the presence of 6 mM BCECF for the internalization of the pH sensitive dye as previously described (49). The lipid suspension underwent 10 freeze-thaw cycles in liquid nitrogen and was extruded through a 100 nm polycarbonate membrane (Millipore). Protein reconstitution was performed by destabilizing the liposomes with 5.5 mM Triton X-100 for 15 min. The protein was added at a final concentration of 15 μg/ml and the mixture was incubated at 4 °C for 15 min. Detergent removal was achieved for 5 h at 4 °C with 25 mg of Bio-Beads SM-2 Resin (Bio-Rad) prehydrated with agitation, repeated 4 times, followed by ultracentrifugation at 16,000*g* for 20 min at 4 °C to harvest liposomes in the pellet. To remove the non-encapsulated dye, the BCECF-containing liposomes were passed through a Sephadex G-25 resin (PD-10 column from GE Healthcare) using 20 mM HEPES, pH 8, 50 mM NaCl, 15% glycerol buffer as eluent. Eluates (500 μl) were collected and analyzed by fluorescence measurement (λ_ex_ 485 nm, λ_em_ 530 nm) and by Western blot using the HRP conjugated anti-His tag antibody to identify fractions containing proteoliposomes with internalized BCECF. The vesicles were then stored in the dark at 4 °C until used.

### C_55_PP phosphatase assay in liposomes

The liposomes were used to measure the C_55_-PP phosphatase activity of BacA in a membrane environment as follows. For each kinetic assay, 5 μl of a reaction mixture containing 20 μM C_15_-PP, 20 μM [^14^C]C_5_-PP, 180 μM C_5_-PP in 20 mM HEPES, pH 7.5, 50 mM NaCl, and 15% glycerol were mixed with 5 μl of protein-free liposomes (as a control) or BacA-containing liposomes. The mixture was then supplemented with UppS at a final concentration of 0.2 mg/ml to initiate the enzymatic synthesis of C_55_-PP. The EntC peptides were added at various concentrations from a water solution. The mixture was incubated at 37 °C for 10 min, and the reaction was then stopped by plunging the tube in liquid nitrogen. Substrates and products were separated and quantified by thin-layer chromatography as previously described for C_55_-PP phosphatase assay in detergent. In that case, the activity was only expressed as relative activity as the absolute concentration of reconstituted protein was not formally determined, the condition without EntC peptides referring to the maximum activity (100%).

### Proteoliposome permeabilisation assay

The membrane permeabilization assay was adapted from already described protocols (49). BCECF-containing liposomes were diluted in a buffer containing 20 mM HEPES, pH 6, 50 mM NaCl, 15% glycerol (20 μl of vesicles in 300 μl of buffer) using black Corning Flat Bottom Polystyrol 96-well plates. The fluorescence was then monitored for 1h with an excitation wavelength of 485 nm and an emission wavelength of 530 nm before, to establish the baseline, and after the addition of EntC at 5 μM. Fluorescence measurements were then normalized based on a value of 1, which was attributed to the last fluorescence value before EntC addition.

### Cross-linking experiments

Crosslinking experiments were performed using the amine-reactive, membrane-permeable, and non-cleavable crosslinker disuccinimidyl suberate (DSS) purchased from Sigma. EntC peptides and BacA protein were mixed at equimolar concentrations (17 μM) in buffer D in the presence of an excess of DSS crosslinker (1000-fold or 100-fold) in a final reaction volume of 20 μl. The reaction was incubated for 30 min at 4°C, and any unreacted DSS was quenched with 50 mM Tris-HCl. After separation on a 12% acrylamide gel, proteins were electrotransferred onto polyvinylidene difluoride (PVDF) membrane, and BacA was detected using peroxidase-coupled anti-His tag antibody (Penta-His HRP conjugate from Qiagen, RRID: AB_3699318) by chemiluminescence detection (Western Lightning Plus-ECL, PerkinElmer) using the ChemiDoc MP system (Bio-Rad).

### Functional complementation tests

The thermosensitive *E. coli* strain BWTetra-Ts*bacA* (12) was transformed by heat shock with plasmids pET:*bacA*_*ef*_ or its mutated variants and the functional complementation test was performed as previously described (27).

### Flow-induced dispersion analysis (FIDA)

The experiments were performed on a FIDA1 instrument equipped with a 480 nm LED-induced fluorescence detector and a FIDA permanently coated capillary (FIDA Biosystems ApS). The sample tray and the capillary chamber were temperature-controlled at 25 °C. The EntC1 peptide was first labelled using the FL 480 Fida1 Protein Labelling Kit according to the manufacturer's instructions. Briefly, the EntC1 peptide was incubated overnight at 4 °C with a 5-fold molar excess of FAM NHS ester reactive dye. The free dye was removed using two successive size exclusion chromatographies, and the purification efficiency was checked using the FIDA1 instrument. The degree of labelling was determined by measuring the absorbance at both 280 nm et 495 nm and was found to be 6%. All protein samples were prepared in the assay buffer 20 mM Tris-HCl, pH 7.4, 200 mM NaCl and 0.1% DDM. All the binding experiments were carried out using the premix method to maintain binding equilibrium during the run. Following the FIDA experimental procedure, the capillary was first rinsed and equilibrated with the assay buffer at 3500 mbar for 2 min. Next, the unlabeled protein was filled at 3500 mbar for 20 s, after which the premix sample (Fluorescein-EntC1 preincubated with the protein) was injected at 50 mbar for 10 s. Finally, the indicator was flowed towards the fluorescence detector with the analyte sample at 400 mbar for 180 s. For binding curves, the FL-EntC1 indicator was fixed at 100 nM and titrated with 0 to 4000 nM BacA_*ef*_ analyte in the absence or presence of 200 nM EntC2 peptide. The Taylorgrams were processed using the FIDA data analysis software (version 2.32) to determine the apparent hydrodynamic radius (Rh) and fit binding curves to the best binding model.

### Isothermal titration microcalorimetry measurements (ITC)

ITC experiments were performed with an ITC200 isothermal titration calorimeter from MicroCal (Malvern Panalytical, Malvern, UK). The experiments were carried out at 20 °C. The protein concentration in the microcalorimeter cell (0.2 ml) was 33 μM. Nineteen 2-μl injections of peptides at 400 μM were performed at intervals of 240 s while stirring at 500 rpm. To limit buffer mismatching effect between the cell and the syringe, and the heat-of-dilution effect for having glycerol in the protein solution, the latter were extensively dialyzed, prior to the ITC measurement, in a buffer containing 20 mM Tris-HCl pH 7.4, 200 mM NaCl, 0.1% DDM, to remove glycerol. Then, the dialysis buffer was used to dilute the EntC peptides to further limit buffer mismatching. The experimental data were fitted to theoretical titration curves with the software supplied by MicroCal (ORIGIN). This software uses the relationship between the heat generated by each injection and ΔH (enthalpy change in kcal/mol), *K*_a_ (the association binding constant in M^−1^), n (the number of binding sites), total protein concentration and free and total ligand concentrations.

### Differential scanning calorimetry (DSC)

Thermal stability of BacA protein in different mixtures of peptides was performed by DSC on a MicroCal model VP-DSC (Malvern Panalytical, Malvern, UK). BacA (33 μM) was mixed with different mixtures of peptides at twice the BacA concentration (*i.e*. 66 μM). Each measurement was preceded by a baseline scan with the standard buffer. Scans were performed at 1 K/min (60 °C/h) temperature ramp between 30 °C and 100 °C. The heat capacity of the buffer was subtracted from that of the protein sample before analysis. The thermodynamic parameters were determined by fitting the data to the following equation:

$$\Delta C_{p}\left(T\right)=K_{d}\left(T\right)\Delta H_{\text{cal}}\Delta H_{\text{vH}}\;/\;{\left[1+K_{d}\left(T\right)\right]}^{2}\;{\text{RT}}^{2}$$

where *K*_d_ is the equilibrium constant for a two-state process, ΔH_vH_ the enthalpy calculated based on a two-state process and ΔH_cal_ the measured enthalpy.

### Alphafold2 modeling

Alphafold2 v2.3 modelling of the 3D protein or complex structures was performed *via* the AlphaFold@I2BC server accessible from the I2BC (Institute for Integrative Biology of the Cell). Multiple Sequence Alignments (MSAs) rely on MMseqs2 (50) while pairwise representation of amino acids is generated using colabfold v1.5.2 (51). Multimeric protein structures were predicted using AlphaFold-Multimer (26). The structural model of the tripartite complex BacA/EntC1/EntC2 is available in ModelArchive at https://modelarchive.org/doi/10.5452/ma-2xesy (52).

## Data availability

The structural model is available in ModelArchive at https://modelarchive.org/doi/10.5452/ma-2xesy.

## Supporting information

This article contains supporting information.

## Conflict of interest

The authors declare that they have no conflicts of interest with the contents of this article.

## Supporting information

Supporting Informations
