# Supplementary material for: Mechanistic insights into enterocin C targeting the undecaprenyl phosphate recycling protein BacA
Source: J Biol Chem. 2025 Nov 11;302(1):110929. doi: 10.1016/j.jbc.2025.110929 (PMC12765074; doi:10.1016/j.jbc.2025.110929)
Supplement: Supporting Informations [file mmc1.pptx]

## Slide 1
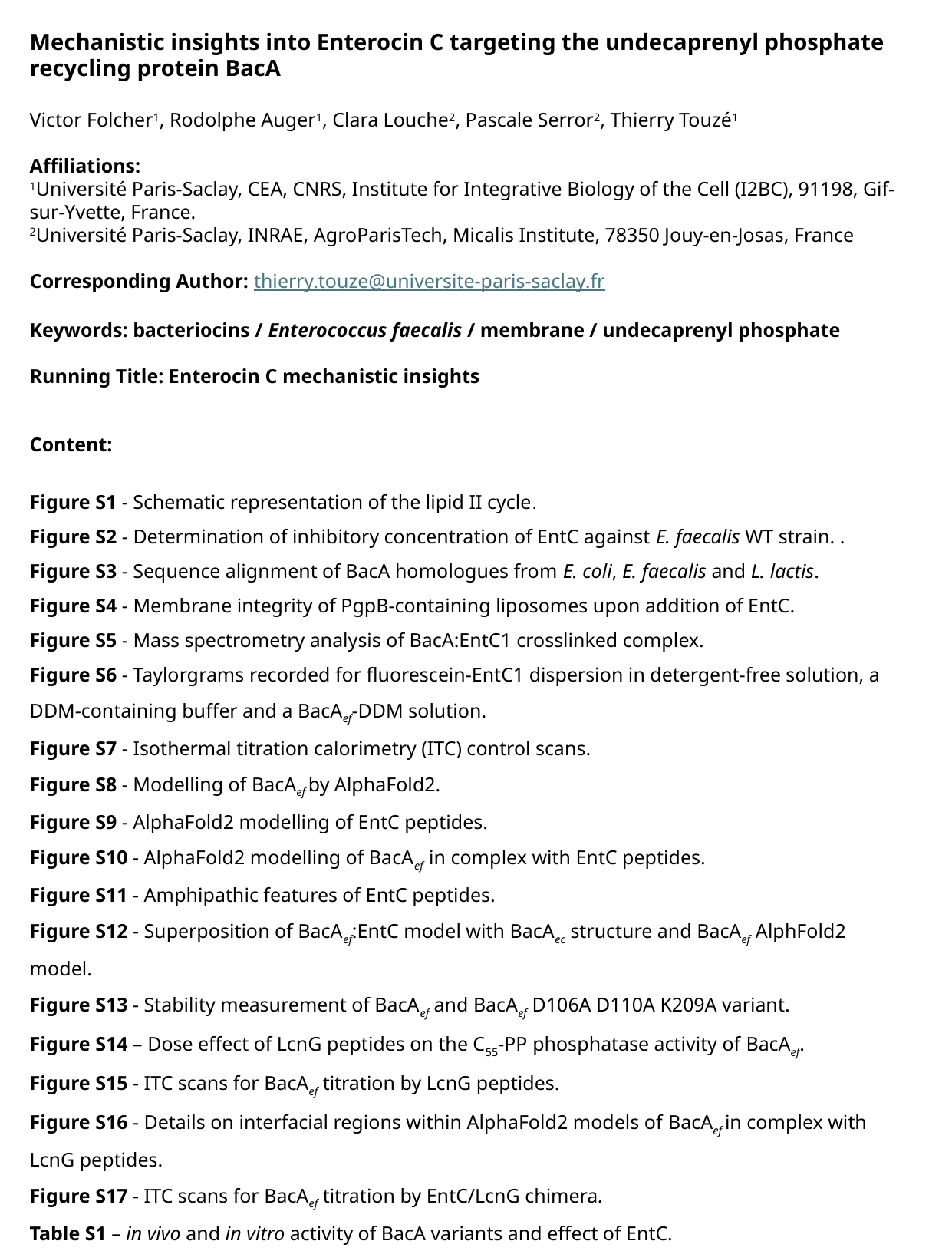

Mechanistic insights into Enterocin C targeting the undecaprenyl phosphate recycling protein BacA
Victor Folcher1, Rodolphe Auger1, Clara Louche2, Pascale Serror2, Thierry Touzé1
Affiliations:
1Université Paris-Saclay, CEA, CNRS, Institute for Integrative Biology of the Cell (I2BC), 91198, Gif-sur-Yvette, France.
2Université Paris-Saclay, INRAE, AgroParisTech, Micalis Institute, 78350 Jouy‑en‑Josas, France
Corresponding Author: thierry.touze@universite-paris-saclay.fr
Keywords: bacteriocins / Enterococcus faecalis / membrane / undecaprenyl phosphate
Running Title: Enterocin C mechanistic insights
Content:
Figure S1 - Schematic representation of the lipid II cycle.
Figure S2 - Determination of inhibitory concentration of EntC against E. faecalis WT strain. .
Figure S3 - Sequence alignment of BacA homologues from E. coli, E. faecalis and L. lactis.
Figure S4 - Membrane integrity of PgpB-containing liposomes upon addition of EntC.
Figure S5 - Mass spectrometry analysis of BacA:EntC1 crosslinked complex.
Figure S6 - Taylorgrams recorded for fluorescein-EntC1 dispersion in detergent-free solution, a DDM-containing buffer and a BacAef-DDM solution.
Figure S7 - Isothermal titration calorimetry (ITC) control scans.
Figure S8 - Modelling of BacAef by AlphaFold2.
Figure S9 - AlphaFold2 modelling of EntC peptides.
Figure S10 - AlphaFold2 modelling of BacAef in complex with EntC peptides.
Figure S11 - Amphipathic features of EntC peptides.
Figure S12 - Superposition of BacAef:EntC model with BacAec structure and BacAef AlphFold2 model.
Figure S13 - Stability measurement of BacAef and BacAef D106A D110A K209A variant.
Figure S14 – Dose effect of LcnG peptides on the C55-PP phosphatase activity of BacAef.
Figure S15 - ITC scans for BacAef titration by LcnG peptides.
Figure S16 - Details on interfacial regions within AlphaFold2 models of BacAef in complex with LcnG peptides.
Figure S17 - ITC scans for BacAef titration by EntC/LcnG chimera.
Table S1 – in vivo and in vitro activity of BacA variants and effect of EntC.
Table S2 – Interfaces within BacAef:EntC tripartite complex according to PISA.
Table S3 – Oligonucleotides used in this study.

## Slide 2
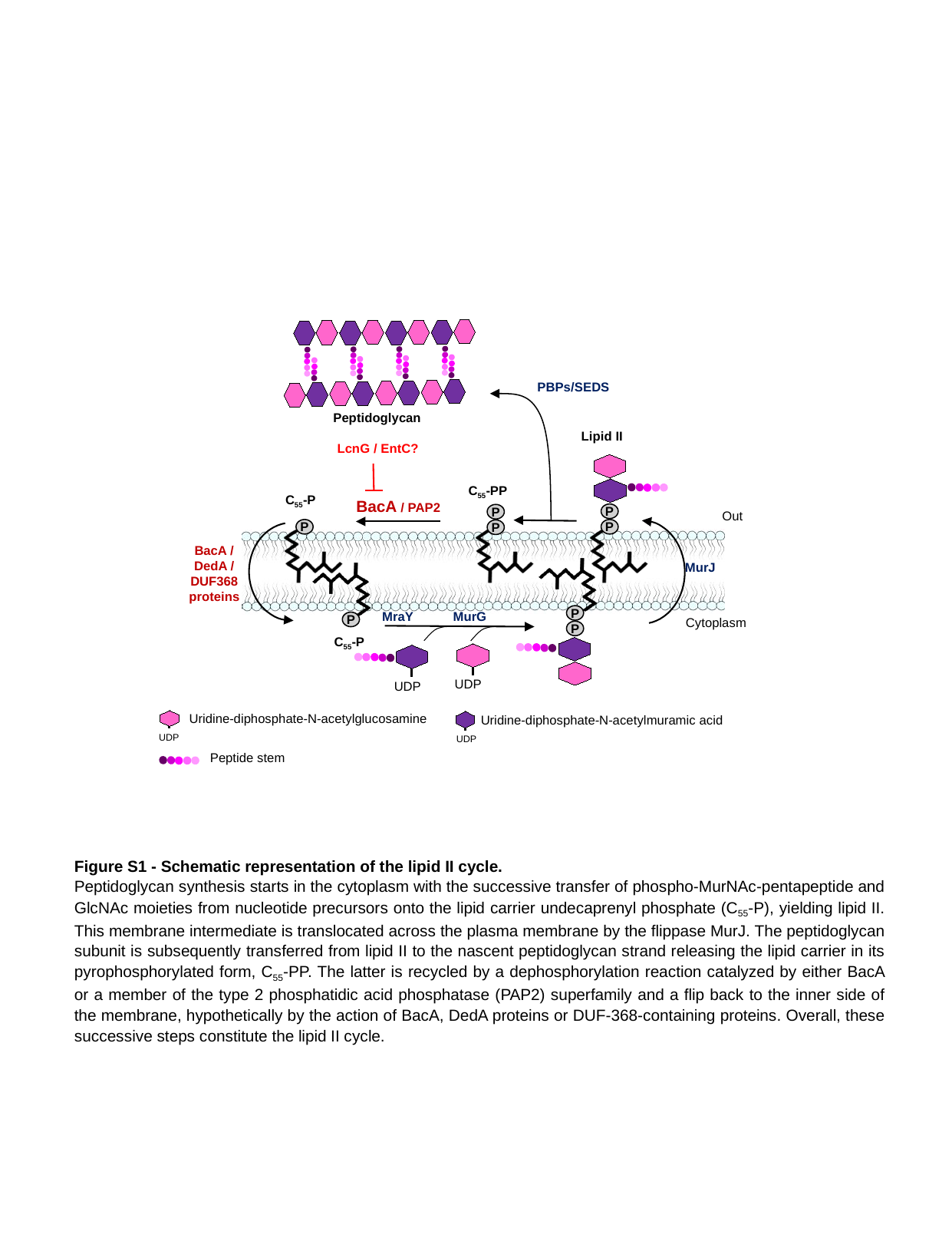

PBPs/SEDS
Peptidoglycan
Lipid II
LcnG / EntC?
C55-PP
C55-P
BacA / PAP2
Out
Cytoplasm
P
P
P
P
P
BacA / DedA / DUF368 proteins
MurJ
MraY MurG
P
P
P
C55-P
UDP
UDP
Uridine-diphosphate-N-acetylglucosamine
Uridine-diphosphate-N-acetylmuramic acid
UDP
UDP
Peptide stem
Figure S1 - Schematic representation of the lipid II cycle.
Peptidoglycan synthesis starts in the cytoplasm with the successive transfer of phospho-MurNAc-pentapeptide and GlcNAc moieties from nucleotide precursors onto the lipid carrier undecaprenyl phosphate (C55-P), yielding lipid II. This membrane intermediate is translocated across the plasma membrane by the flippase MurJ. The peptidoglycan subunit is subsequently transferred from lipid II to the nascent peptidoglycan strand releasing the lipid carrier in its pyrophosphorylated form, C55-PP. The latter is recycled by a dephosphorylation reaction catalyzed by either BacA or a member of the type 2 phosphatidic acid phosphatase (PAP2) superfamily and a flip back to the inner side of the membrane, hypothetically by the action of BacA, DedA proteins or DUF-368-containing proteins. Overall, these successive steps constitute the lipid II cycle.

## Slide 3
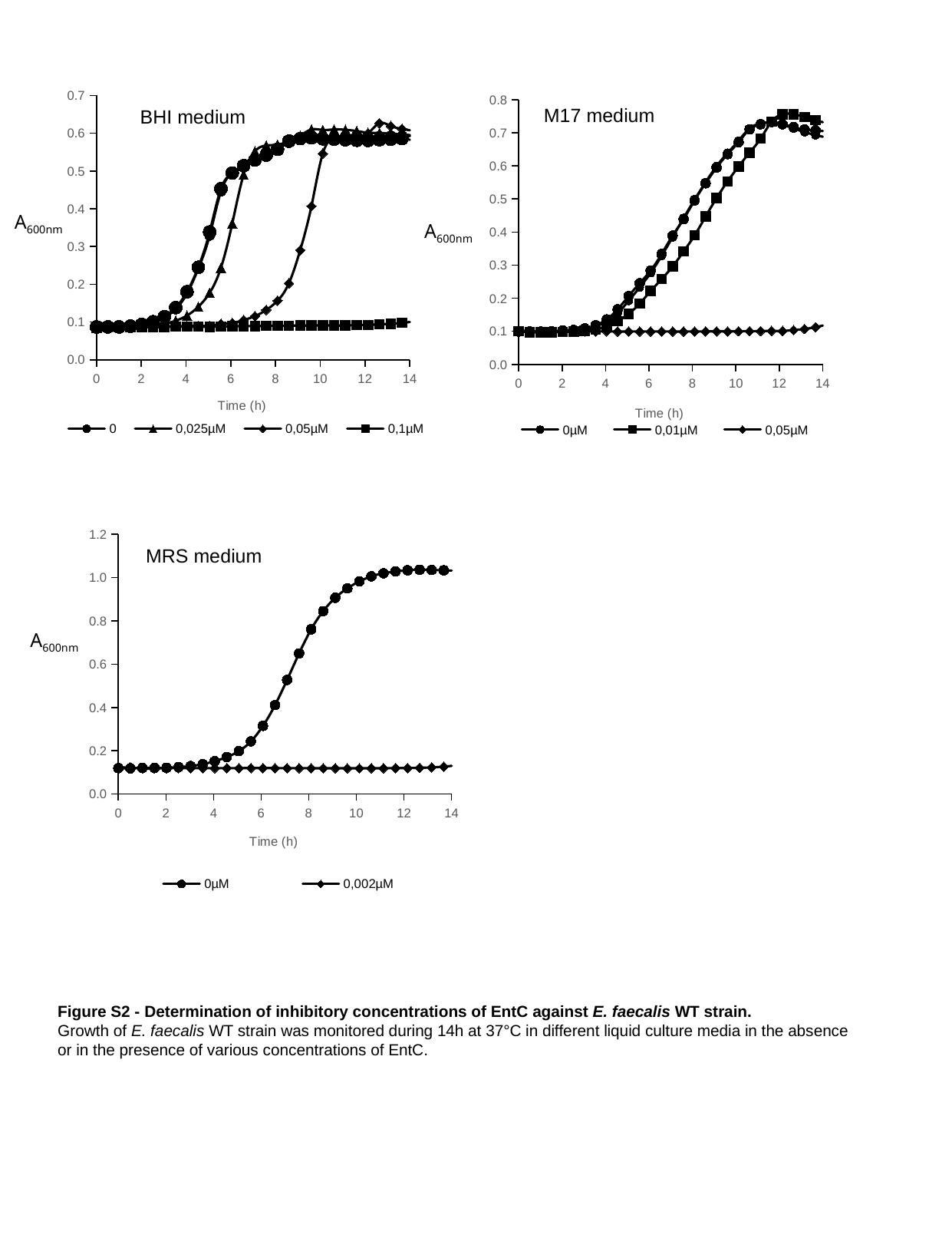

### Chart
| Category | 0µM | 0µM | 0,01µM | 0,05µM |
|---|---|---|---|---|M17 medium
### Chart
| Category | 0 | 0 | 0,025µM | 0,05µM | 0,1µM |
|---|---|---|---|---|---|BHI medium
A600nm
A600nm
### Chart
| Category | 0µM | 0,002µM |
|---|---|---|MRS medium
A600nm
Figure S2 - Determination of inhibitory concentrations of EntC against E. faecalis WT strain.
Growth of E. faecalis WT strain was monitored during 14h at 37°C in different liquid culture media in the absence or in the presence of various concentrations of EntC.

## Slide 4
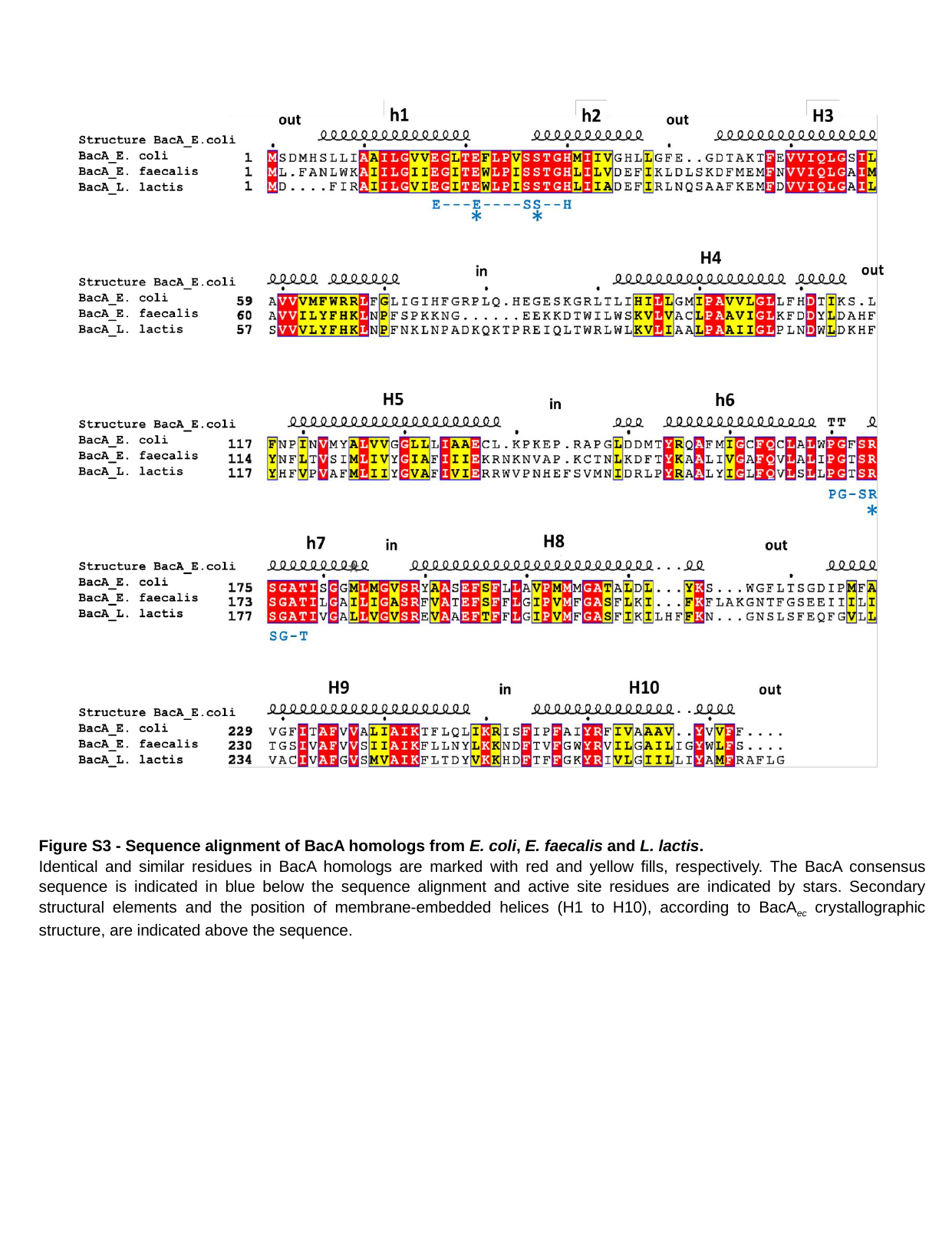

Figure S3 - Sequence alignment of BacA homologs from E. coli, E. faecalis and L. lactis.
Identical and similar residues in BacA homologs are marked with red and yellow fills, respectively. The BacA consensus sequence is indicated in blue below the sequence alignment and active site residues are indicated by stars. Secondary structural elements and the position of membrane-embedded helices (H1 to H10), according to BacAec crystallographic structure, are indicated above the sequence.

## Slide 5
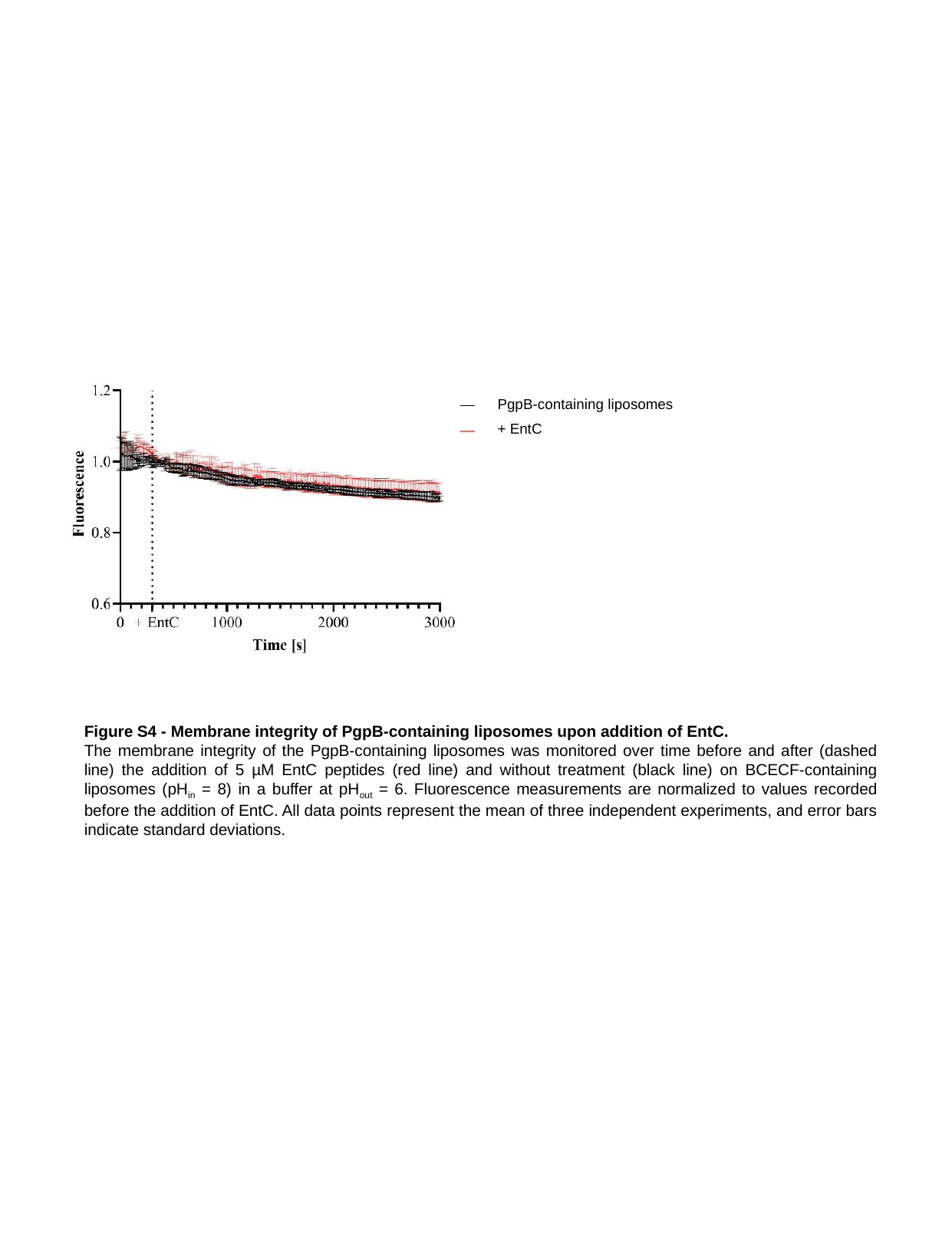

PgpB-containing liposomes
+ EntC
Figure S4 - Membrane integrity of PgpB-containing liposomes upon addition of EntC.
The membrane integrity of the PgpB-containing liposomes was monitored over time before and after (dashed line) the addition of 5 µM EntC peptides (red line) and without treatment (black line) on BCECF-containing liposomes (pHin = 8) in a buffer at pHout = 6. Fluorescence measurements are normalized to values recorded before the addition of EntC. All data points represent the mean of three independent experiments, and error bars indicate standard deviations.

## Slide 6
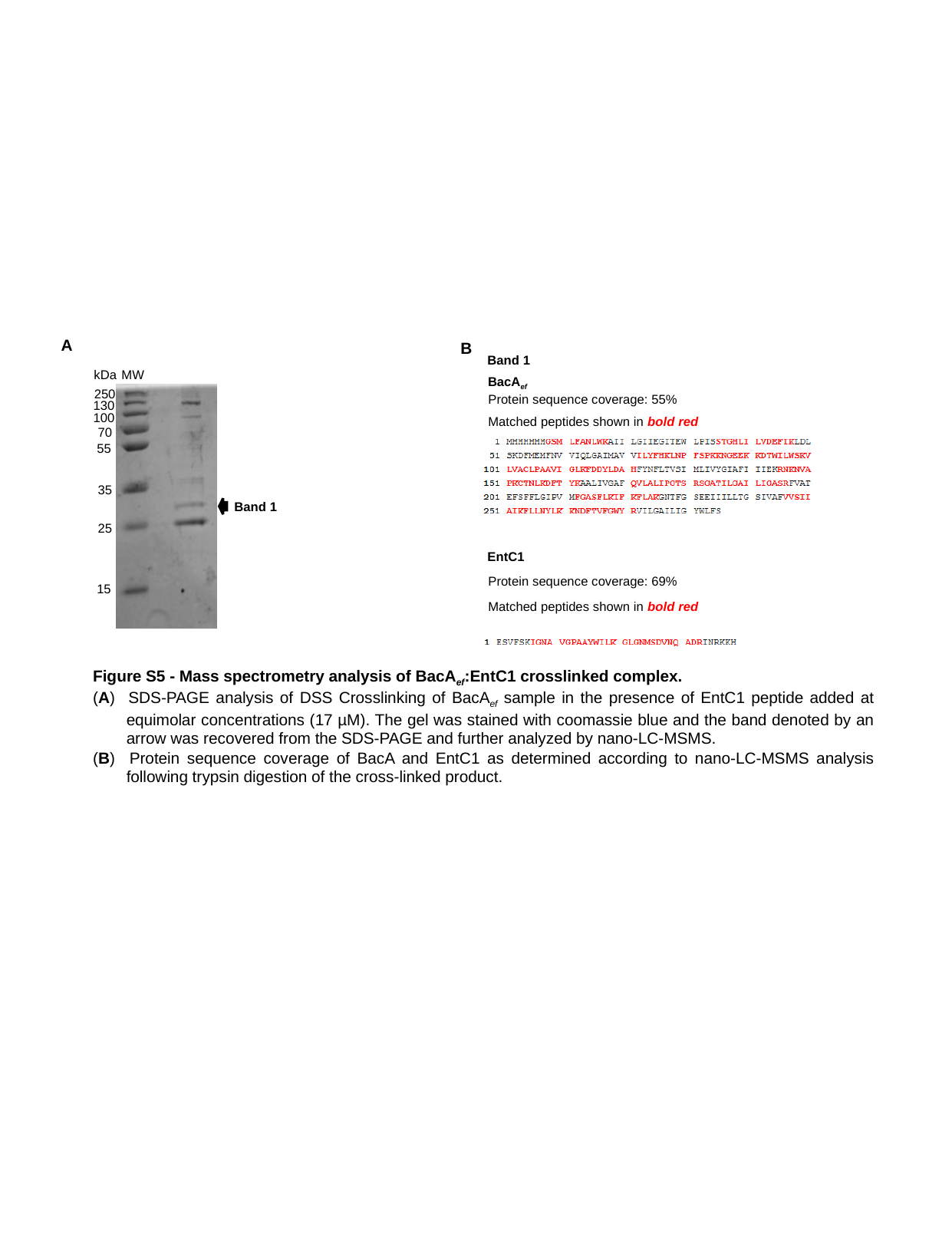

A
B
Band 1
kDa
MW
250
130
100
70
55
35
25
15
BacAef
Protein sequence coverage: 55%
Matched peptides shown in bold red
Band 1
EntC1
Protein sequence coverage: 69%
Matched peptides shown in bold red
Figure S5 - Mass spectrometry analysis of BacAef:EntC1 crosslinked complex.
(A) 	SDS-PAGE analysis of DSS Crosslinking of BacAef sample in the presence of EntC1 peptide added at equimolar concentrations (17 µM). The gel was stained with coomassie blue and the band denoted by an arrow was recovered from the SDS-PAGE and further analyzed by nano-LC-MSMS.
(B) 	Protein sequence coverage of BacA and EntC1 as determined according to nano-LC-MSMS analysis following trypsin digestion of the cross-linked product.

## Slide 7
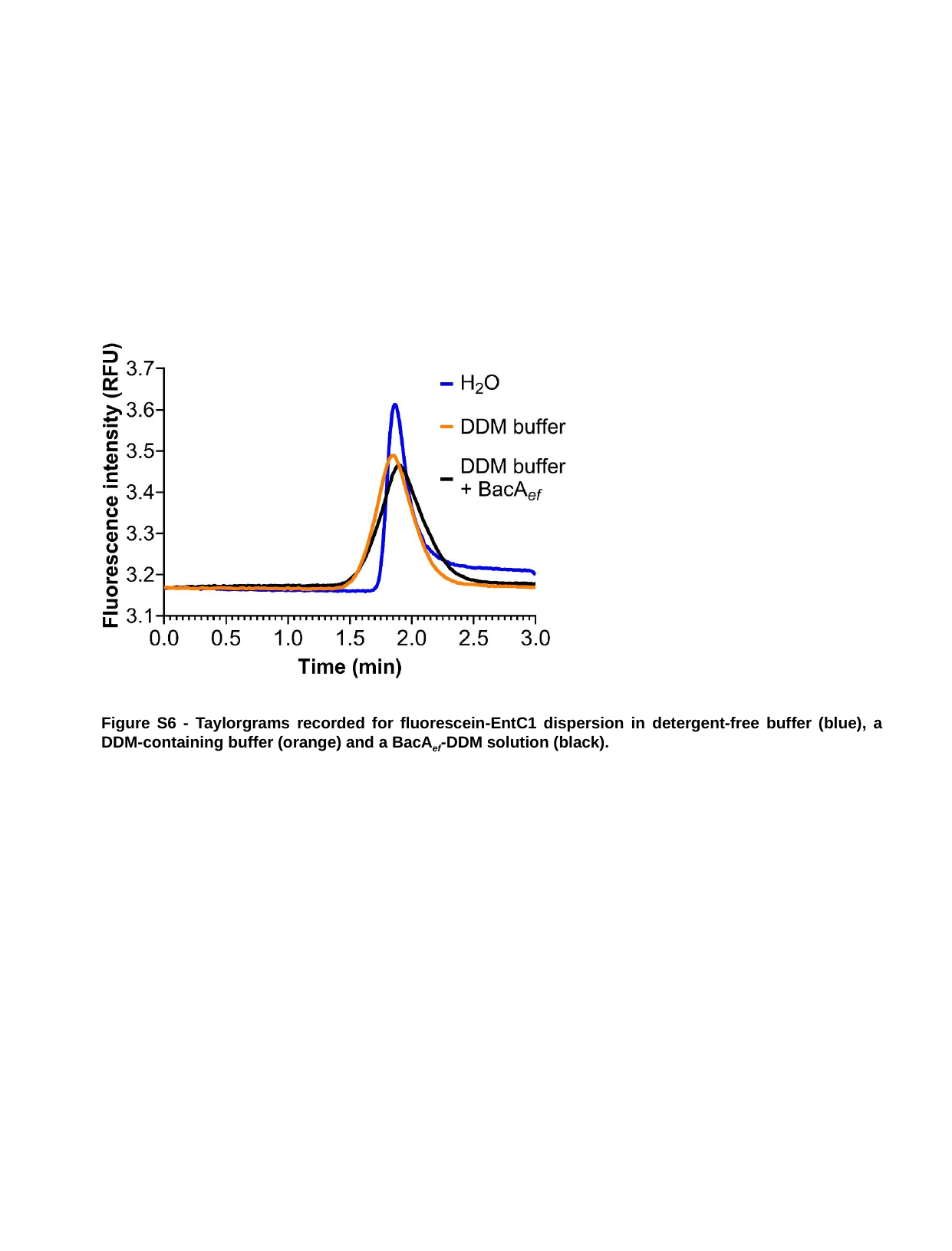

Figure S6 - Taylorgrams recorded for fluorescein-EntC1 dispersion in detergent-free buffer (blue), a DDM-containing buffer (orange) and a BacAef-DDM solution (black).

## Slide 8
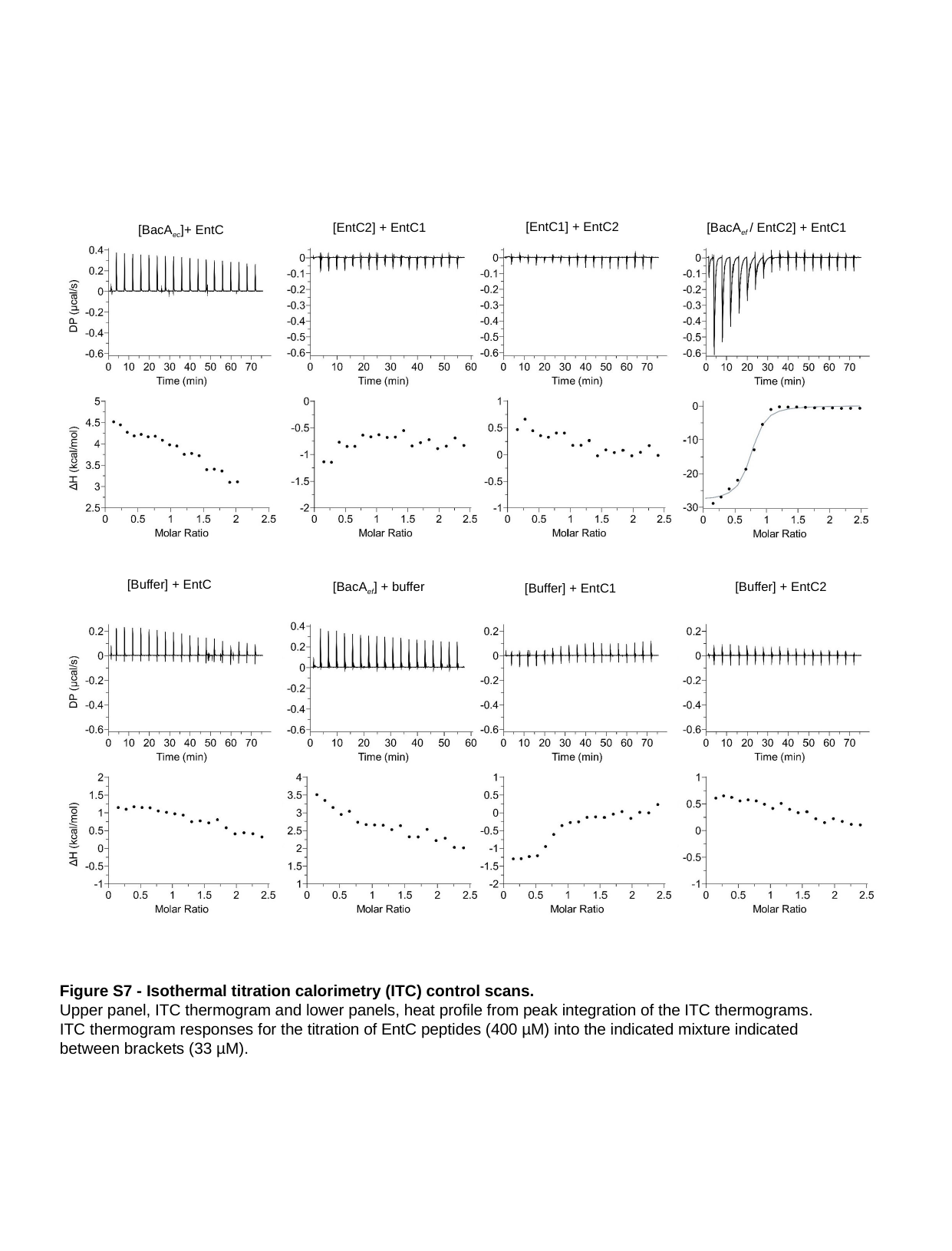

[EntC1] + EntC2
[BacAef / EntC2] + EntC1
[EntC2] + EntC1
[BacAec]+ EntC
No binding
[Buffer] + EntC
[BacAef] + buffer
[Buffer] + EntC2
[Buffer] + EntC1
Figure S7 - Isothermal titration calorimetry (ITC) control scans.
Upper panel, ITC thermogram and lower panels, heat profile from peak integration of the ITC thermograms.
ITC thermogram responses for the titration of EntC peptides (400 µM) into the indicated mixture indicated between brackets (33 µM).

## Slide 9
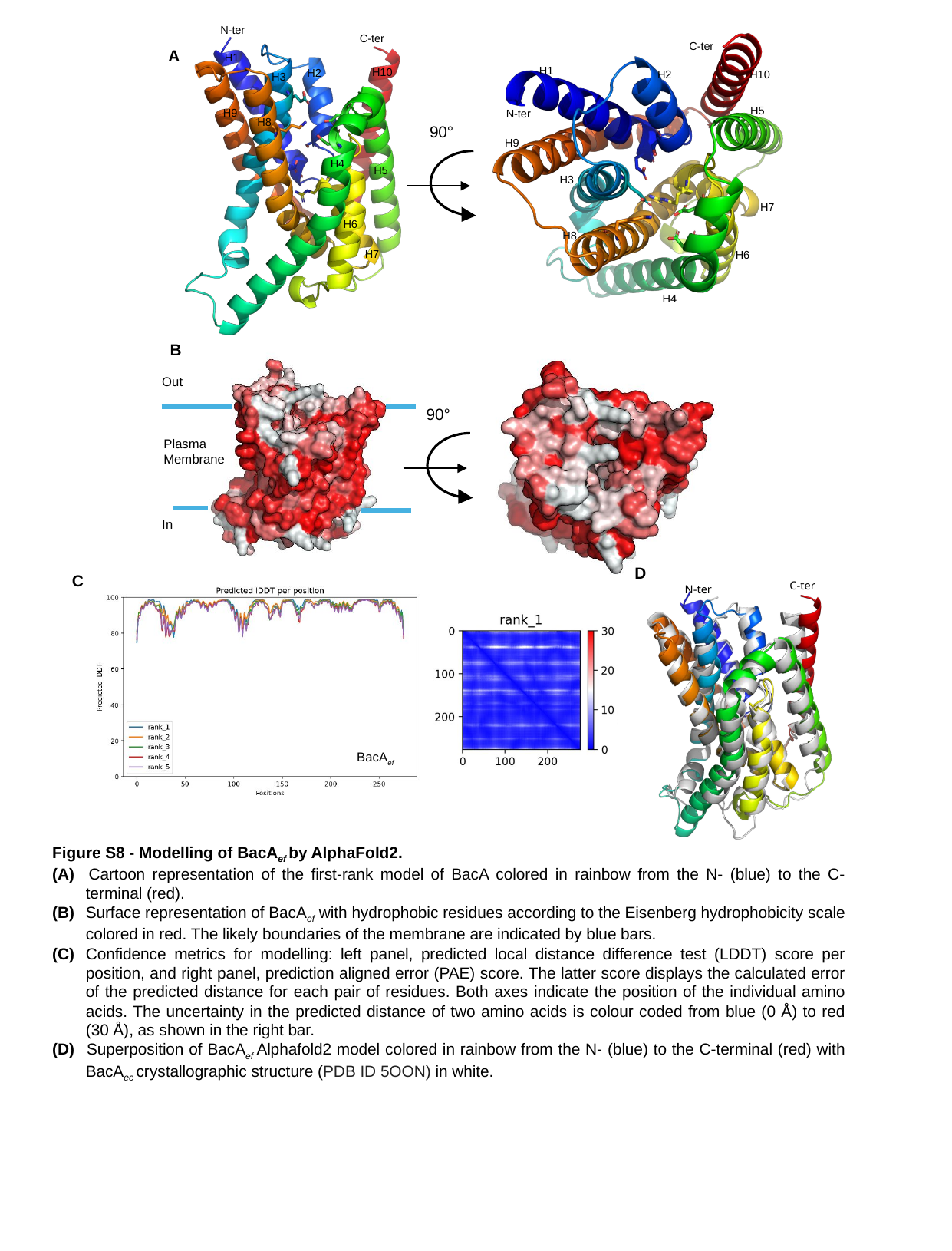

N-ter
C-ter
H1
H10
H2
H3
H9
H8
H4
H5
H6
H7
C-ter
H1
H2
H10
H5
N-ter
H9
H3
H7
H8
H6
H4
A
90°
B
Out
90°
Plasma
Membrane
In
D
C
C-ter
N-ter
BacAef
Figure S8 - Modelling of BacAef by AlphaFold2.
(A) 	Cartoon representation of the first-rank model of BacA colored in rainbow from the N- (blue) to the C-terminal (red).
(B) 	Surface representation of BacAef with hydrophobic residues according to the Eisenberg hydrophobicity scale colored in red. The likely boundaries of the membrane are indicated by blue bars.
(C)	Confidence metrics for modelling: left panel, predicted local distance difference test (LDDT) score per position, and right panel, prediction aligned error (PAE) score. The latter score displays the calculated error of the predicted distance for each pair of residues. Both axes indicate the position of the individual amino acids. The uncertainty in the predicted distance of two amino acids is colour coded from blue (0 Å) to red (30 Å), as shown in the right bar.
(D) 	Superposition of BacAef Alphafold2 model colored in rainbow from the N- (blue) to the C-terminal (red) with BacAec crystallographic structure (PDB ID 5OON) in white.

## Slide 10
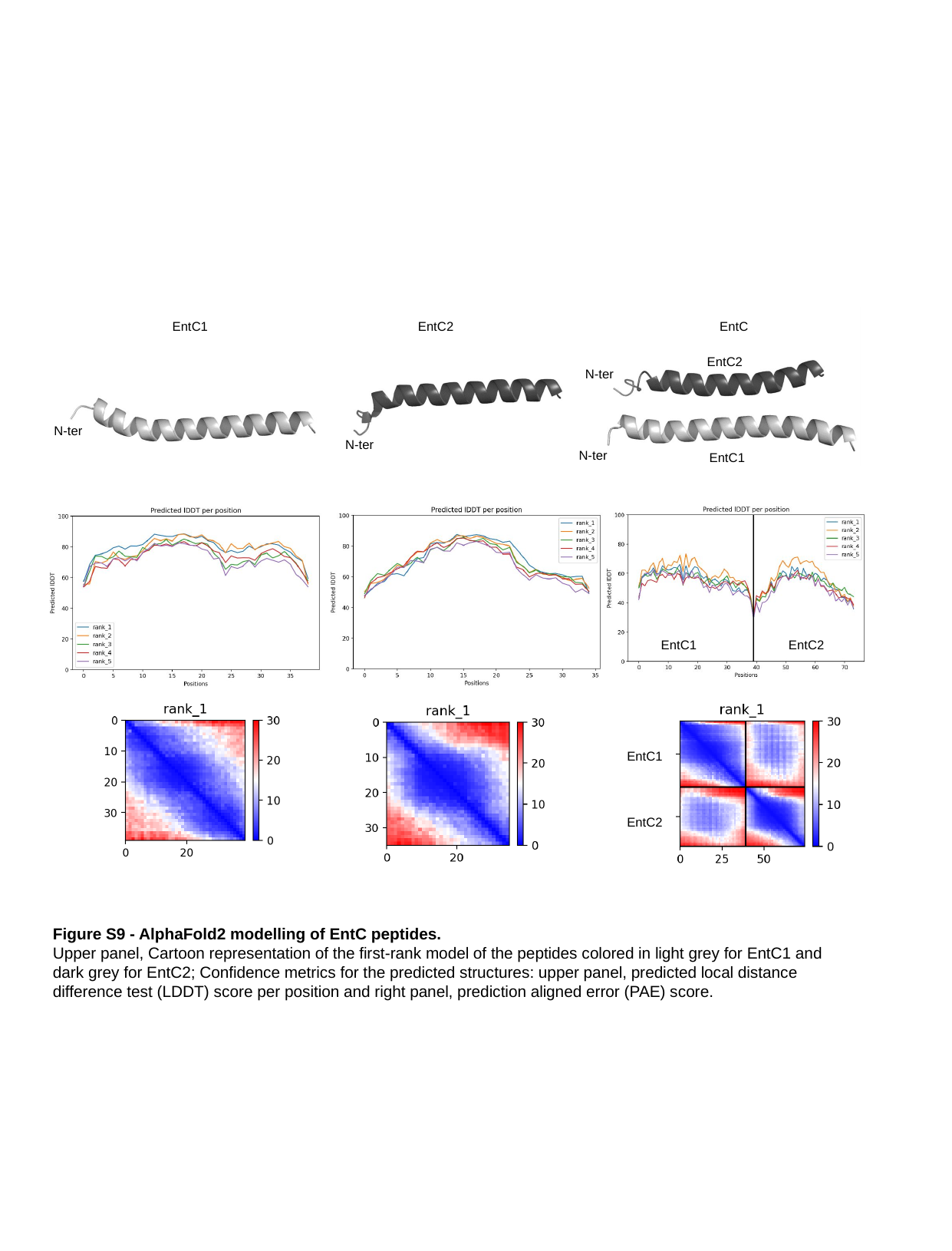

EntC1
EntC2
EntC
EntC2
N-ter
N-ter
N-ter
N-ter
EntC1
EntC1
EntC2
EntC1
EntC2
Figure S9 - AlphaFold2 modelling of EntC peptides.
Upper panel, Cartoon representation of the first-rank model of the peptides colored in light grey for EntC1 and dark grey for EntC2; Confidence metrics for the predicted structures: upper panel, predicted local distance difference test (LDDT) score per position and right panel, prediction aligned error (PAE) score.

## Slide 11
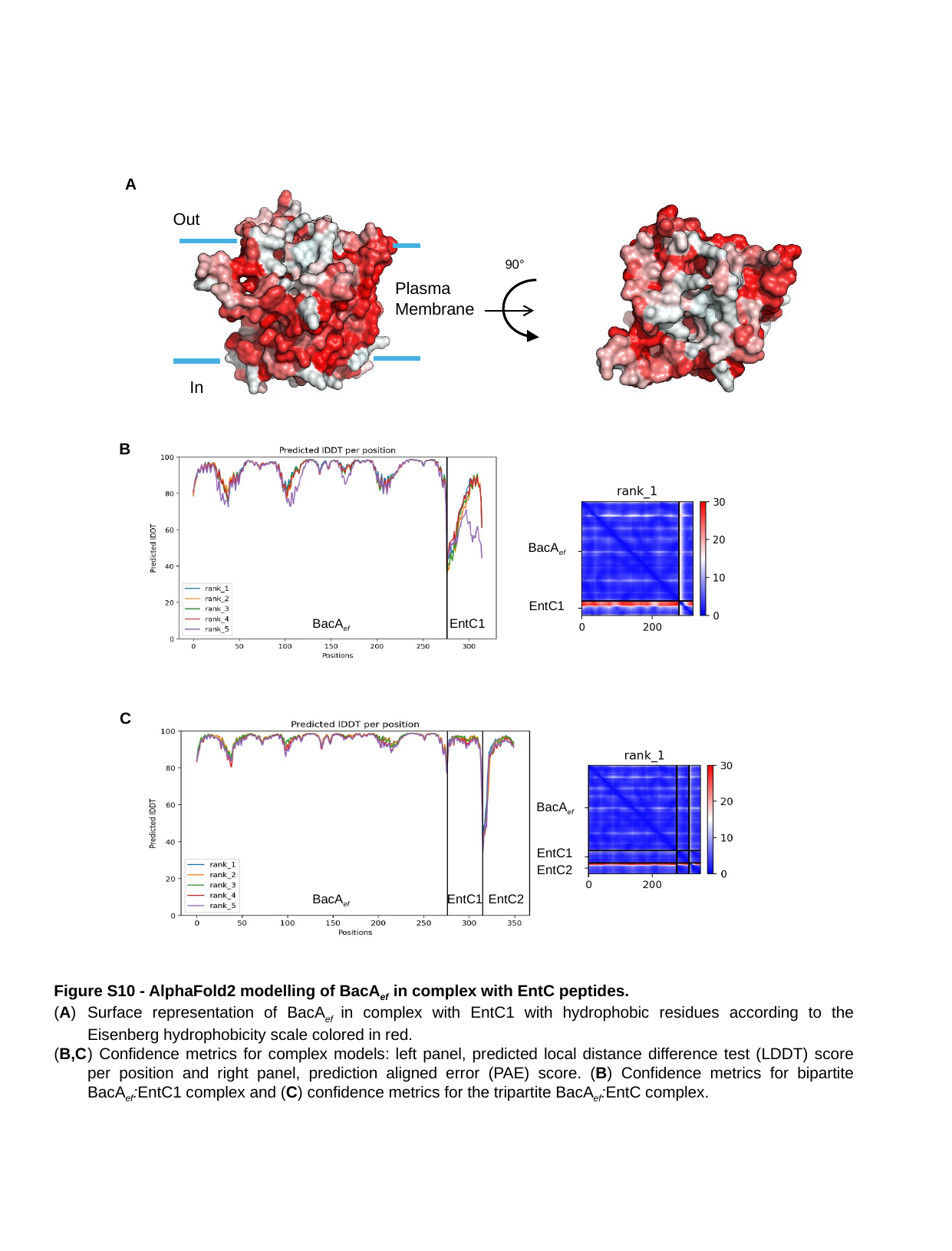

A
Out
Plasma
Membrane
In
90°
B
BacAef
EntC1
BacAef
EntC1
C
BacAef
EntC1
EntC2
BacAef
EntC1
EntC2
Figure S10 - AlphaFold2 modelling of BacAef in complex with EntC peptides.
(A)	Surface representation of BacAef in complex with EntC1 with hydrophobic residues according to the Eisenberg hydrophobicity scale colored in red.
(B,C	) Confidence metrics for complex models: left panel, predicted local distance difference test (LDDT) score per position and right panel, prediction aligned error (PAE) score. (B) Confidence metrics for bipartite BacAef:EntC1 complex and (C) confidence metrics for the tripartite BacAef:EntC complex.

## Slide 12
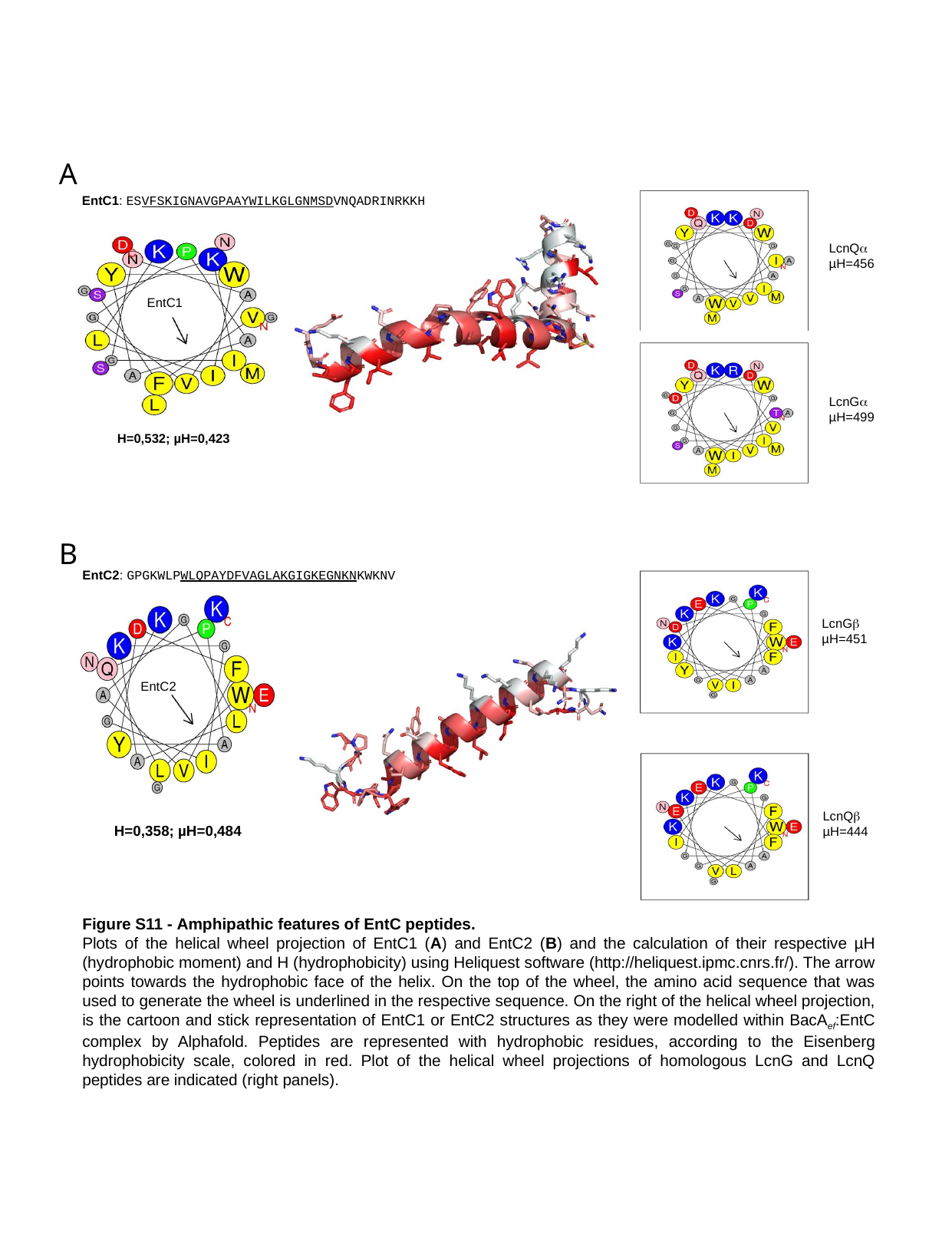

A
B
EntC1: ESVFSKIGNAVGPAAYWILKGLGNMSDVNQADRINRKKH
LcnQa
µH=456
EntC1
LcnGa
µH=499
H=0,532; µH=0,423
EntC2: GPGKWLPWLQPAYDFVAGLAKGIGKEGNKNKWKNV
LcnGb
µH=451
EntC2
LcnQb
µH=444
H=0,358; µH=0,484
Figure S11 - Amphipathic features of EntC peptides.
Plots of the helical wheel projection of EntC1 (A) and EntC2 (B) and the calculation of their respective µH (hydrophobic moment) and H (hydrophobicity) using Heliquest software (http://heliquest.ipmc.cnrs.fr/). The arrow points towards the hydrophobic face of the helix. On the top of the wheel, the amino acid sequence that was used to generate the wheel is underlined in the respective sequence. On the right of the helical wheel projection, is the cartoon and stick representation of EntC1 or EntC2 structures as they were modelled within BacAef:EntC complex by Alphafold. Peptides are represented with hydrophobic residues, according to the Eisenberg hydrophobicity scale, colored in red. Plot of the helical wheel projections of homologous LcnG and LcnQ peptides are indicated (right panels).

## Slide 13
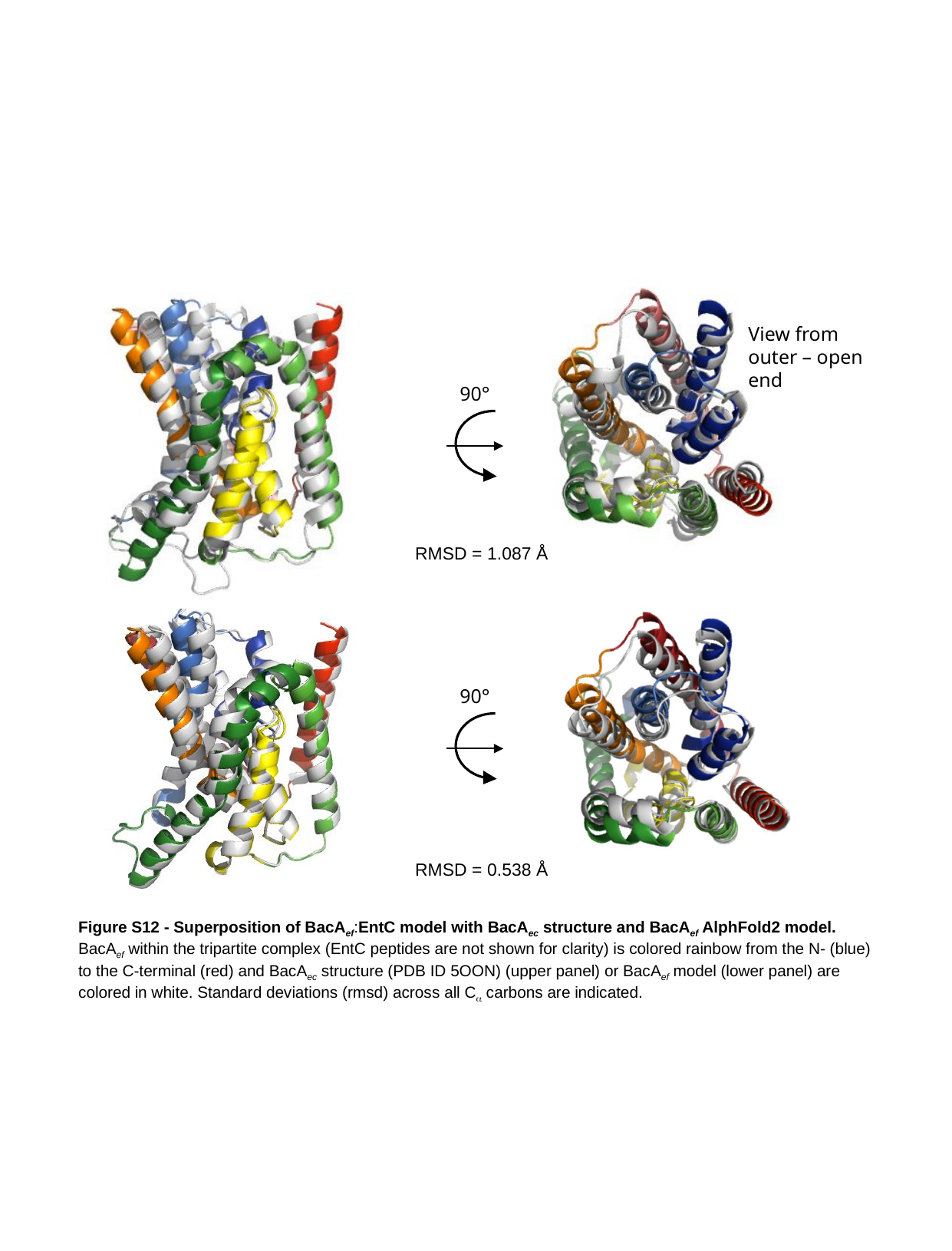

View from outer – open end
90°
RMSD = 1.087 Å
90°
RMSD = 0.538 Å
Figure S12 - Superposition of BacAef:EntC model with BacAec structure and BacAef AlphFold2 model.
BacAef within the tripartite complex (EntC peptides are not shown for clarity) is colored rainbow from the N- (blue) to the C-terminal (red) and BacAec structure (PDB ID 5OON) (upper panel) or BacAef model (lower panel) are colored in white. Standard deviations (rmsd) across all Ca carbons are indicated.

## Slide 14
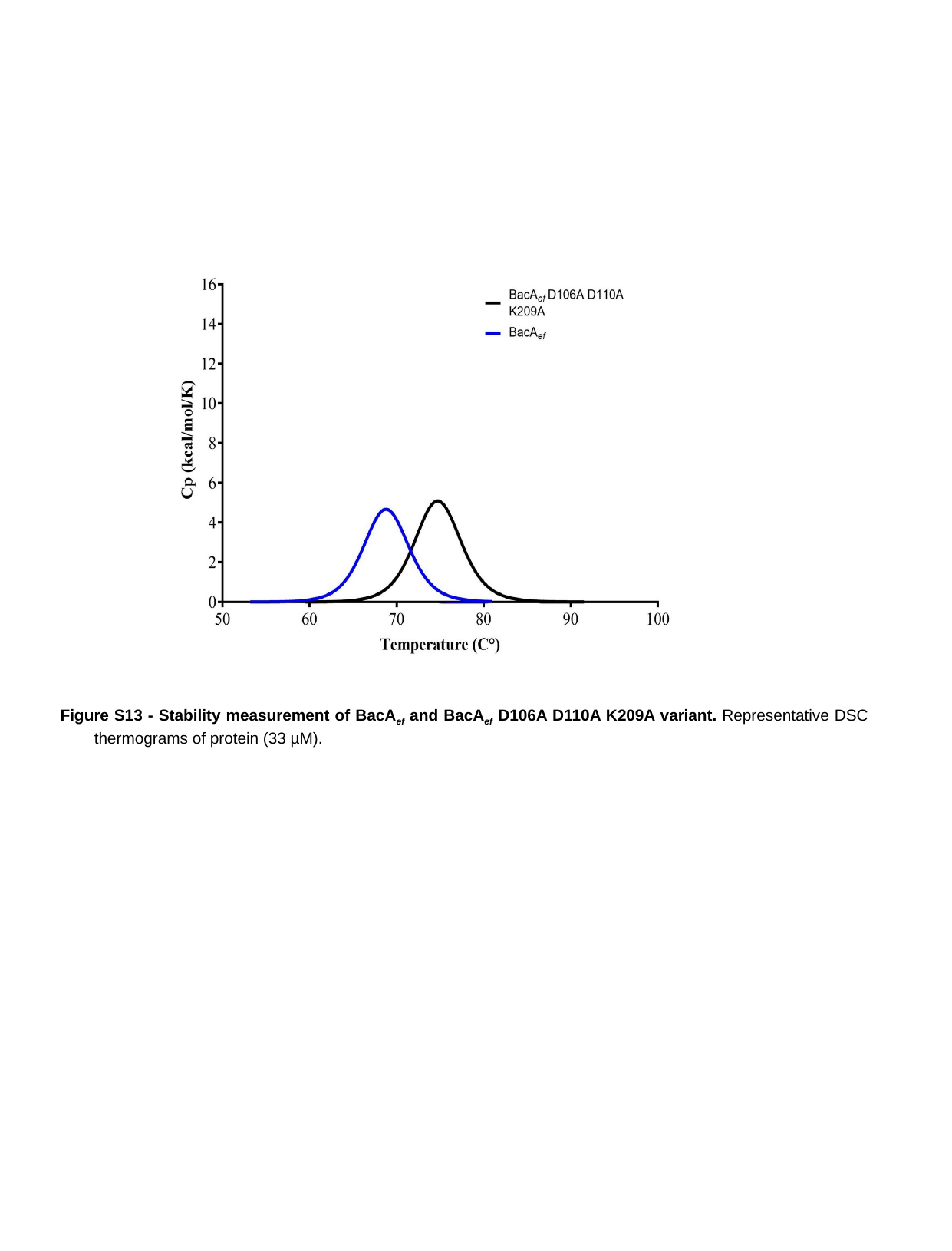

Figure S13 - Stability measurement of BacAef and BacAef D106A D110A K209A variant. Representative DSC thermograms of protein (33 µM).

## Slide 15
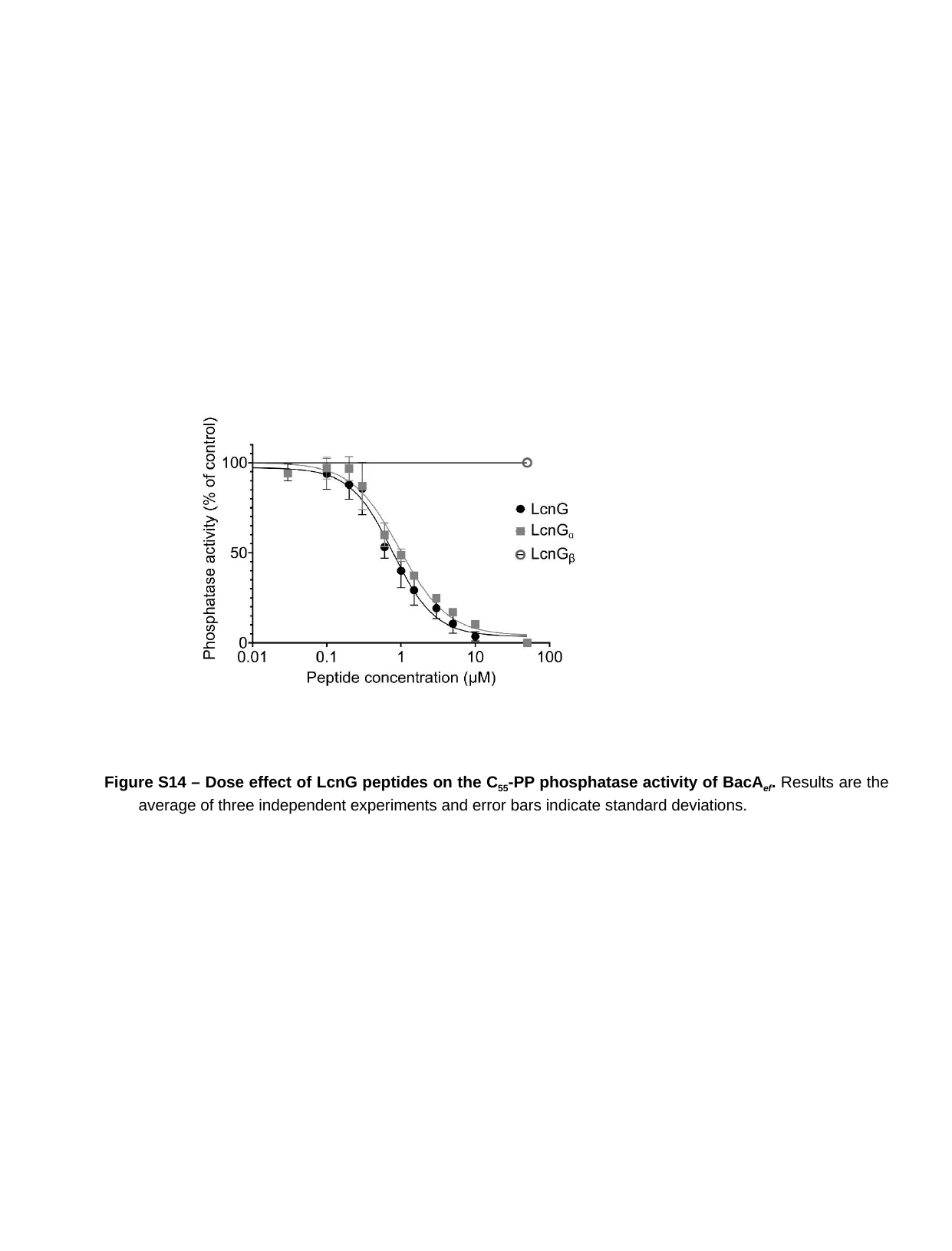

Figure S14 – Dose effect of LcnG peptides on the C55-PP phosphatase activity of BacAef. Results are the average of three independent experiments and error bars indicate standard deviations.

## Slide 16
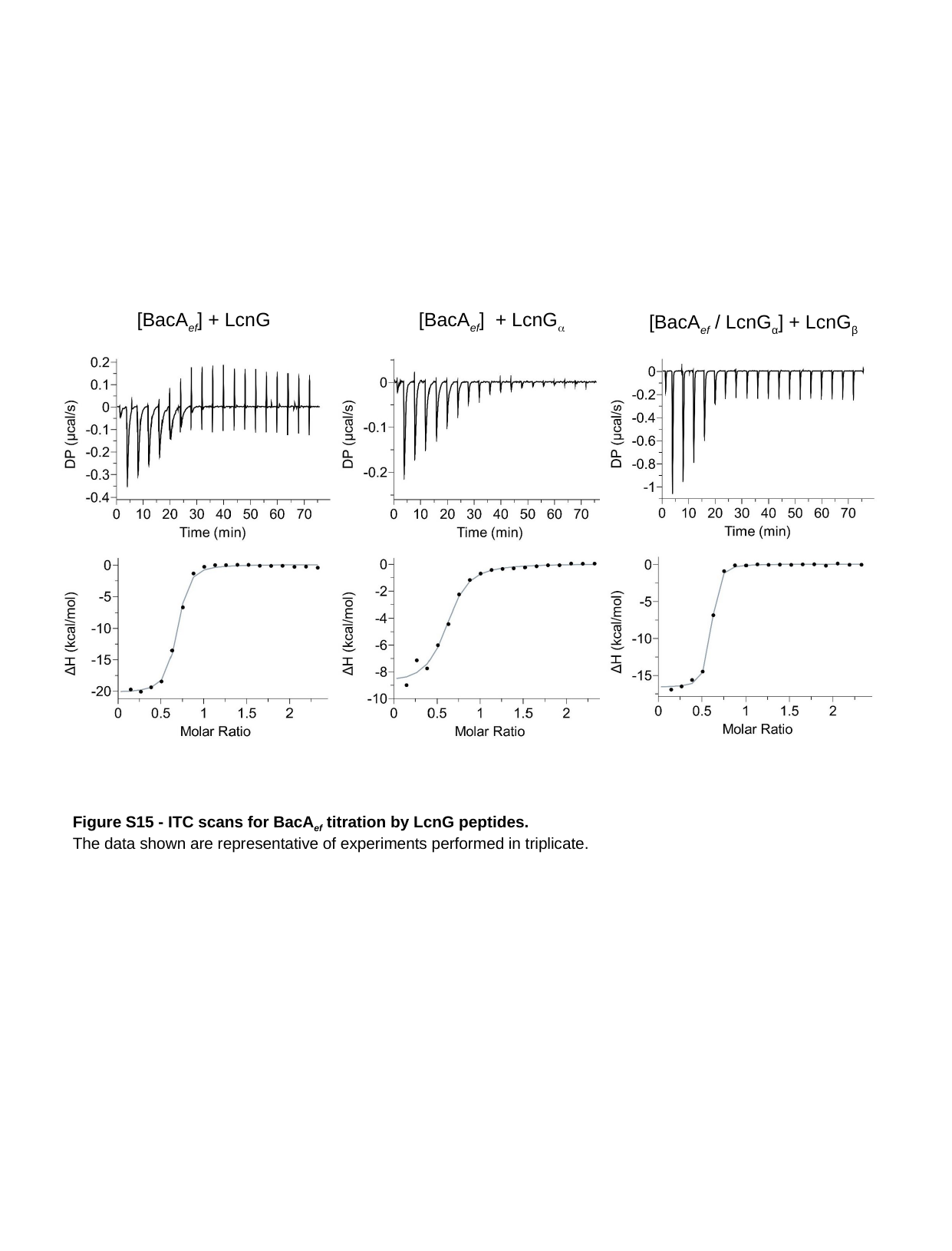

[BacAef] + LcnG
[BacAef] + LcnGa
[BacAef / LcnGα] + LcnGβ
Figure S15 - ITC scans for BacAef titration by LcnG peptides.
The data shown are representative of experiments performed in triplicate.

## Slide 17
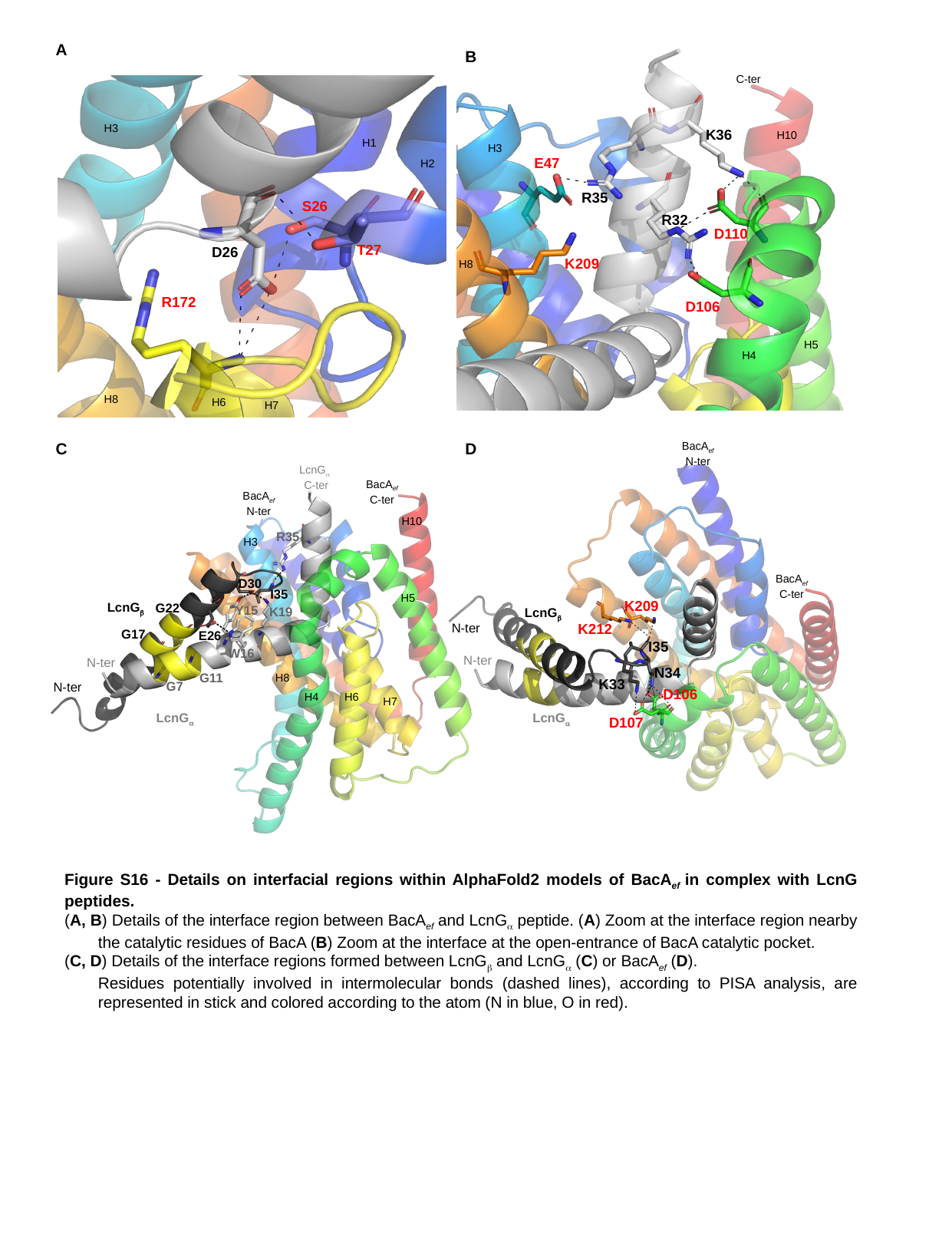

C-ter
K36
H10
H3
E47
R35
R32
D110
K209
H8
D106
H5
H4
A
B
H3
H1
H2
S26
T27
D26
R172
H8
H6
H7
K209
LcnGb
K212
I35
N34
K33
D106
LcnGa
D107
BacAef
N-ter
LcnGa
C-ter
BacAef
C-ter
BacAef
N-ter
H10
R35
H3
BacAef
C-ter
D30
I35
H5
LcnGb
G22
Y15
K19
N-ter
G17
E26
W16
N-ter
N-ter
G11
H8
G7
N-ter
H4
H6
H7
LcnGa
C
D
Figure S16 - Details on interfacial regions within AlphaFold2 models of BacAef in complex with LcnG peptides.
(A, B) Details of the interface region between BacAef and LcnGa peptide. (A) Zoom at the interface region nearby the catalytic residues of BacA (B) Zoom at the interface at the open-entrance of BacA catalytic pocket.
(C, D) Details of the interface regions formed between LcnGb and LcnGa (C) or BacAef (D).
	Residues potentially involved in intermolecular bonds (dashed lines), according to PISA analysis, are represented in stick and colored according to the atom (N in blue, O in red).

## Slide 18
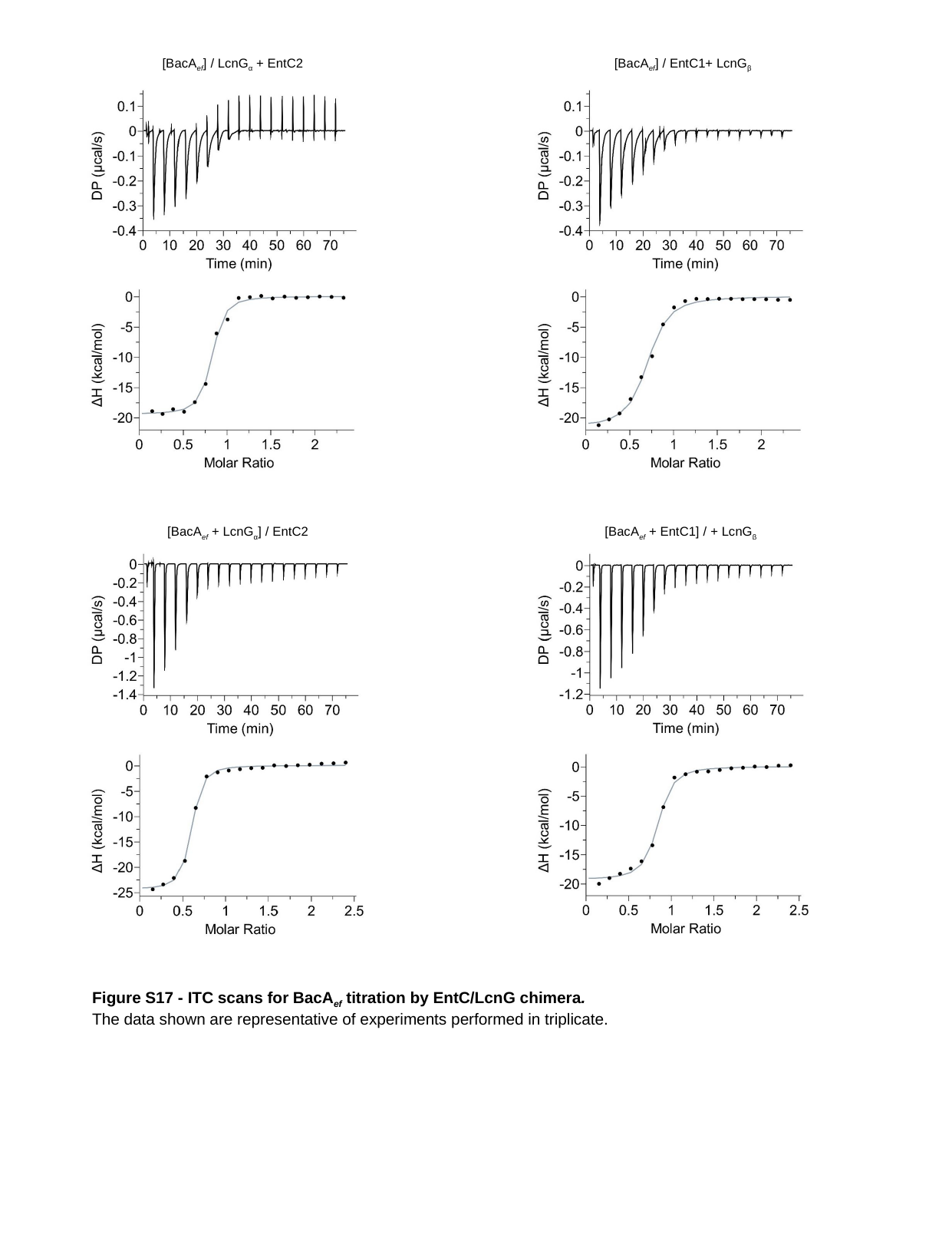

[BacAef] / LcnGα + EntC2
[BacAef] / EntC1+ LcnGβ
[BacAef + LcnGα] / EntC2
[BacAef + EntC1] / + LcnGβ
Figure S17 - ITC scans for BacAef titration by EntC/LcnG chimera.
The data shown are representative of experiments performed in triplicate.

## Slide 19
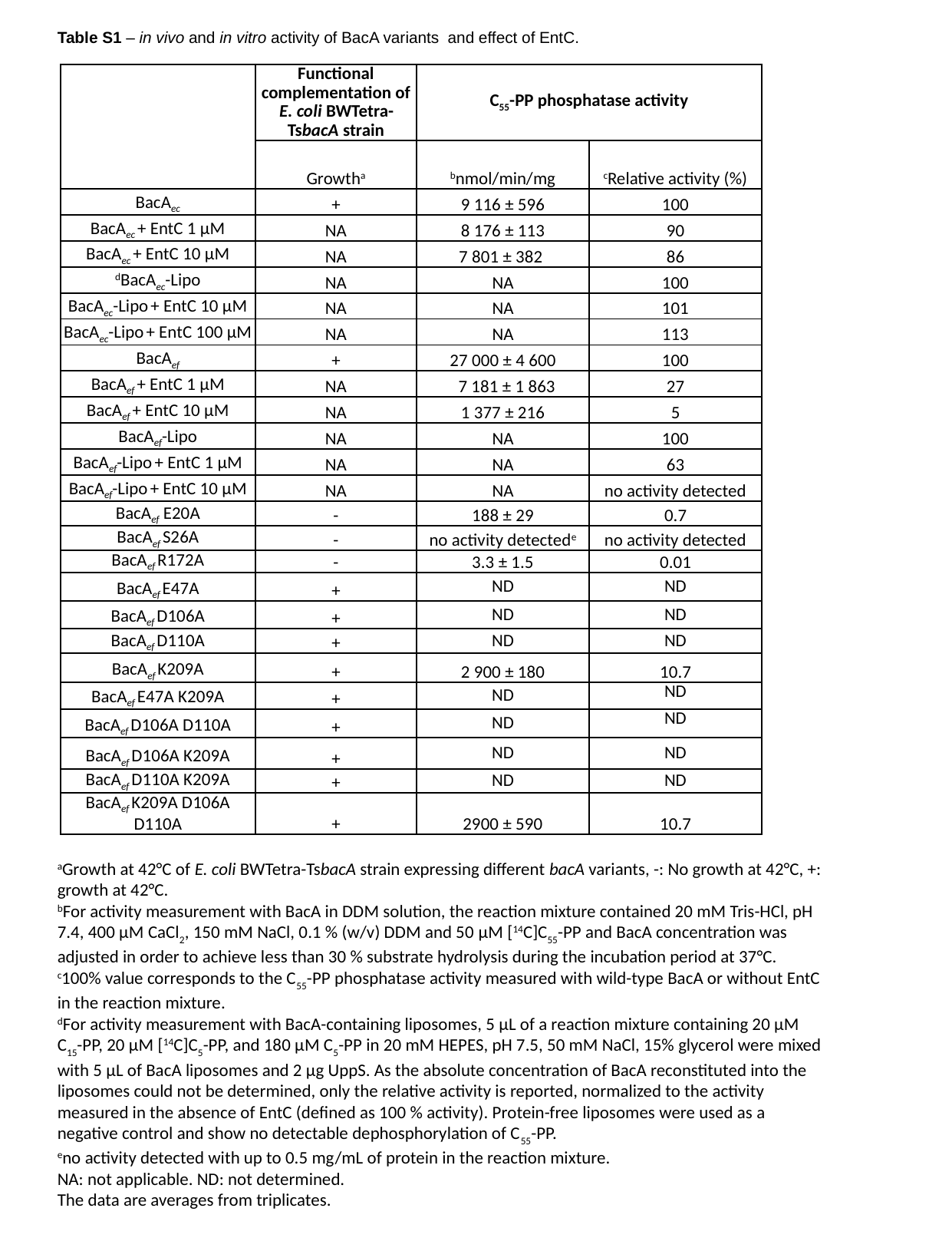

Table S1 – in vivo and in vitro activity of BacA variants and effect of EntC.
| | Functional complementation of E. coli BWTetra-TsbacA strain | C55-PP phosphatase activity | |
| --- | --- | --- | --- |
| | Growtha | bnmol/min/mg | cRelative activity (%) |
| BacAec | + | 9 116 ± 596 | 100 |
| BacAec + EntC 1 µM | NA | 8 176 ± 113 | 90 |
| BacAec + EntC 10 µM | NA | 7 801 ± 382 | 86 |
| dBacAec-Lipo | NA | NA | 100 |
| BacAec-Lipo + EntC 10 µM | NA | NA | 101 |
| BacAec-Lipo + EntC 100 µM | NA | NA | 113 |
| BacAef | + | 27 000 ± 4 600 | 100 |
| BacAef + EntC 1 µM | NA | 7 181 ± 1 863 | 27 |
| BacAef + EntC 10 µM | NA | 1 377 ± 216 | 5 |
| BacAef-Lipo | NA | NA | 100 |
| BacAef-Lipo + EntC 1 µM | NA | NA | 63 |
| BacAef-Lipo + EntC 10 µM | NA | NA | no activity detected |
| BacAef E20A | - | 188 ± 29 | 0.7 |
| BacAef S26A | - | no activity detectede | no activity detected |
| BacAef R172A | - | 3.3 ± 1.5 | 0.01 |
| BacAef E47A | + | ND | ND |
| BacAef D106A | + | ND | ND |
| BacAef D110A | + | ND | ND |
| BacAef K209A | + | 2 900 ± 180 | 10.7 |
| BacAef E47A K209A | + | ND | ND |
| BacAef D106A D110A | + | ND | ND |
| BacAef D106A K209A | + | ND | ND |
| BacAef D110A K209A | + | ND | ND |
| BacAef K209A D106A D110A | + | 2900 ± 590 | 10.7 |
aGrowth at 42°C of E. coli BWTetra-TsbacA strain expressing different bacA variants, -: No growth at 42°C, +: growth at 42°C.
bFor activity measurement with BacA in DDM solution, the reaction mixture contained 20 mM Tris-HCl, pH 7.4, 400 µM CaCl2, 150 mM NaCl, 0.1 % (w/v) DDM and 50 μM [14C]C55-PP and BacA concentration was adjusted in order to achieve less than 30 % substrate hydrolysis during the incubation period at 37°C.
c100% value corresponds to the C55-PP phosphatase activity measured with wild-type BacA or without EntC in the reaction mixture.
dFor activity measurement with BacA-containing liposomes, 5 µL of a reaction mixture containing 20 µM C15-PP, 20 µM [14C]C5-PP, and 180 µM C5-PP in 20 mM HEPES, pH 7.5, 50 mM NaCl, 15% glycerol were mixed with 5 µL of BacA liposomes and 2 µg UppS. As the absolute concentration of BacA reconstituted into the liposomes could not be determined, only the relative activity is reported, normalized to the activity measured in the absence of EntC (defined as 100 % activity). Protein-free liposomes were used as a negative control and show no detectable dephosphorylation of C55-PP.
eno activity detected with up to 0.5 mg/mL of protein in the reaction mixture.
NA: not applicable. ND: not determined.
The data are averages from triplicates.

## Slide 20
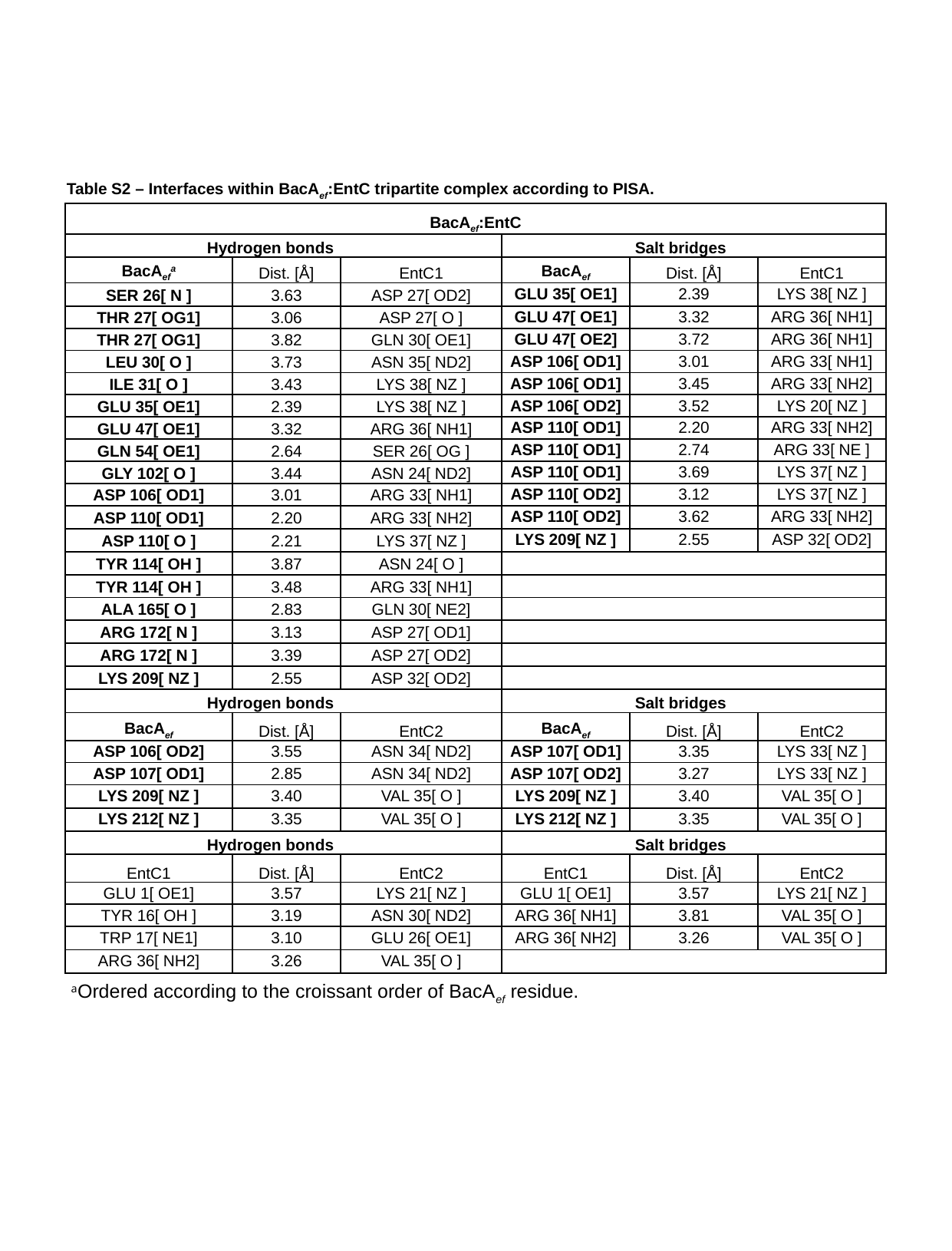

Table S2 – Interfaces within BacAef:EntC tripartite complex according to PISA.
| BacAef:EntC | | | | | |
| --- | --- | --- | --- | --- | --- |
| Hydrogen bonds | | | Salt bridges | | |
| BacAefa | Dist. [Å] | EntC1 | BacAef | Dist. [Å] | EntC1 |
| SER 26[ N ] | 3.63 | ASP 27[ OD2] | GLU 35[ OE1] | 2.39 | LYS 38[ NZ ] |
| THR 27[ OG1] | 3.06 | ASP 27[ O ] | GLU 47[ OE1] | 3.32 | ARG 36[ NH1] |
| THR 27[ OG1] | 3.82 | GLN 30[ OE1] | GLU 47[ OE2] | 3.72 | ARG 36[ NH1] |
| LEU 30[ O ] | 3.73 | ASN 35[ ND2] | ASP 106[ OD1] | 3.01 | ARG 33[ NH1] |
| ILE 31[ O ] | 3.43 | LYS 38[ NZ ] | ASP 106[ OD1] | 3.45 | ARG 33[ NH2] |
| GLU 35[ OE1] | 2.39 | LYS 38[ NZ ] | ASP 106[ OD2] | 3.52 | LYS 20[ NZ ] |
| GLU 47[ OE1] | 3.32 | ARG 36[ NH1] | ASP 110[ OD1] | 2.20 | ARG 33[ NH2] |
| GLN 54[ OE1] | 2.64 | SER 26[ OG ] | ASP 110[ OD1] | 2.74 | ARG 33[ NE ] |
| GLY 102[ O ] | 3.44 | ASN 24[ ND2] | ASP 110[ OD1] | 3.69 | LYS 37[ NZ ] |
| ASP 106[ OD1] | 3.01 | ARG 33[ NH1] | ASP 110[ OD2] | 3.12 | LYS 37[ NZ ] |
| ASP 110[ OD1] | 2.20 | ARG 33[ NH2] | ASP 110[ OD2] | 3.62 | ARG 33[ NH2] |
| ASP 110[ O ] | 2.21 | LYS 37[ NZ ] | LYS 209[ NZ ] | 2.55 | ASP 32[ OD2] |
| TYR 114[ OH ] | 3.87 | ASN 24[ O ] | | | |
| TYR 114[ OH ] | 3.48 | ARG 33[ NH1] | | | |
| ALA 165[ O ] | 2.83 | GLN 30[ NE2] | | | |
| ARG 172[ N ] | 3.13 | ASP 27[ OD1] | | | |
| ARG 172[ N ] | 3.39 | ASP 27[ OD2] | | | |
| LYS 209[ NZ ] | 2.55 | ASP 32[ OD2] | | | |
| Hydrogen bonds | | | Salt bridges | | |
| BacAef | Dist. [Å] | EntC2 | BacAef | Dist. [Å] | EntC2 |
| ASP 106[ OD2] | 3.55 | ASN 34[ ND2] | ASP 107[ OD1] | 3.35 | LYS 33[ NZ ] |
| ASP 107[ OD1] | 2.85 | ASN 34[ ND2] | ASP 107[ OD2] | 3.27 | LYS 33[ NZ ] |
| LYS 209[ NZ ] | 3.40 | VAL 35[ O ] | LYS 209[ NZ ] | 3.40 | VAL 35[ O ] |
| LYS 212[ NZ ] | 3.35 | VAL 35[ O ] | LYS 212[ NZ ] | 3.35 | VAL 35[ O ] |
| Hydrogen bonds | | | Salt bridges | | |
| EntC1 | Dist. [Å] | EntC2 | EntC1 | Dist. [Å] | EntC2 |
| GLU 1[ OE1] | 3.57 | LYS 21[ NZ ] | GLU 1[ OE1] | 3.57 | LYS 21[ NZ ] |
| TYR 16[ OH ] | 3.19 | ASN 30[ ND2] | ARG 36[ NH1] | 3.81 | VAL 35[ O ] |
| TRP 17[ NE1] | 3.10 | GLU 26[ OE1] | ARG 36[ NH2] | 3.26 | VAL 35[ O ] |
| ARG 36[ NH2] | 3.26 | VAL 35[ O ] | | | |
aOrdered according to the croissant order of BacAef residue.

## Slide 21
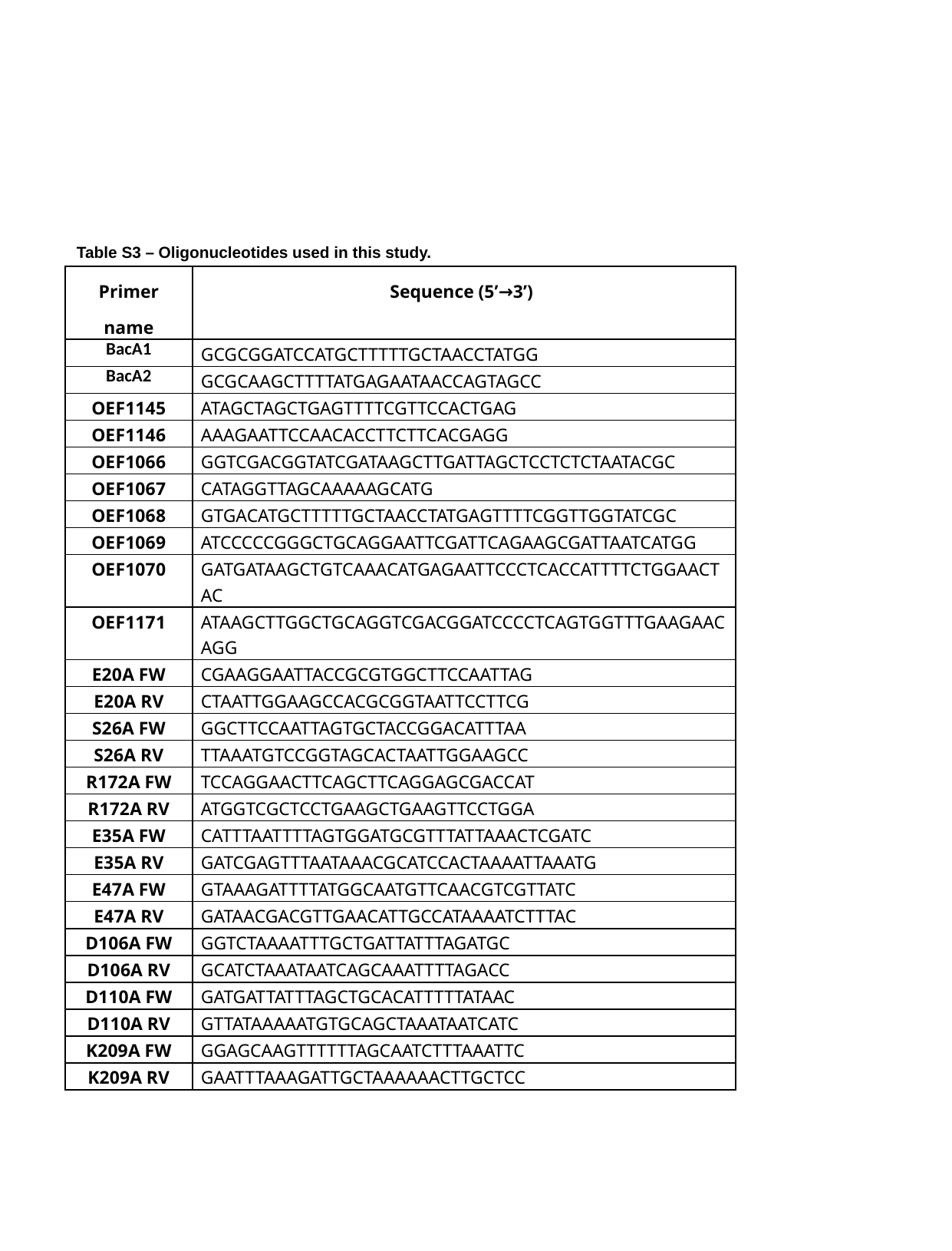

Table S3 – Oligonucleotides used in this study.
| Primer name | Sequence (5’→3’) |
| --- | --- |
| BacA1 | GCGCGGATCCATGCTTTTTGCTAACCTATGG |
| BacA2 | GCGCAAGCTTTTATGAGAATAACCAGTAGCC |
| OEF1145 | ATAGCTAGCTGAGTTTTCGTTCCACTGAG |
| OEF1146 | AAAGAATTCCAACACCTTCTTCACGAGG |
| OEF1066 | GGTCGACGGTATCGATAAGCTTGATTAGCTCCTCTCTAATACGC |
| OEF1067 | CATAGGTTAGCAAAAAGCATG |
| OEF1068 | GTGACATGCTTTTTGCTAACCTATGAGTTTTCGGTTGGTATCGC |
| OEF1069 | ATCCCCCGGGCTGCAGGAATTCGATTCAGAAGCGATTAATCATGG |
| OEF1070 | GATGATAAGCTGTCAAACATGAGAATTCCCTCACCATTTTCTGGAACTAC |
| OEF1171 | ATAAGCTTGGCTGCAGGTCGACGGATCCCCTCAGTGGTTTGAAGAACAGG |
| E20A FW | CGAAGGAATTACCGCGTGGCTTCCAATTAG |
| E20A RV | CTAATTGGAAGCCACGCGGTAATTCCTTCG |
| S26A FW | GGCTTCCAATTAGTGCTACCGGACATTTAA |
| S26A RV | TTAAATGTCCGGTAGCACTAATTGGAAGCC |
| R172A FW | TCCAGGAACTTCAGCTTCAGGAGCGACCAT |
| R172A RV | ATGGTCGCTCCTGAAGCTGAAGTTCCTGGA |
| E35A FW | CATTTAATTTTAGTGGATGCGTTTATTAAACTCGATC |
| E35A RV | GATCGAGTTTAATAAACGCATCCACTAAAATTAAATG |
| E47A FW | GTAAAGATTTTATGGCAATGTTCAACGTCGTTATC |
| E47A RV | GATAACGACGTTGAACATTGCCATAAAATCTTTAC |
| D106A FW | GGTCTAAAATTTGCTGATTATTTAGATGC |
| D106A RV | GCATCTAAATAATCAGCAAATTTTAGACC |
| D110A FW | GATGATTATTTAGCTGCACATTTTTATAAC |
| D110A RV | GTTATAAAAATGTGCAGCTAAATAATCATC |
| K209A FW | GGAGCAAGTTTTTTAGCAATCTTTAAATTC |
| K209A RV | GAATTTAAAGATTGCTAAAAAACTTGCTCC |
